# TAL Effector Repertoires of Strains of *Xanthomonas phaseoli* pv. *manihotis* in Commercial Cassava Crops Reveal High Diversity at the Country Scale

**DOI:** 10.3390/microorganisms9020315

**Published:** 2021-02-04

**Authors:** Carlos A. Zárate-Chaves, Daniela Osorio-Rodríguez, Rubén E. Mora, Álvaro L. Pérez-Quintero, Alexis Dereeper, Silvia Restrepo, Camilo E. López, Boris Szurek, Adriana Bernal

**Affiliations:** 1 PHIM, CIRAD, INRAe, IRD, Montpellier SupAgro, University of Montpellier, 34090 Montpellier, France; carlos.zarate-chaves@ird.fr (C.A.Z.-C.); alperezqui@gmail.com (Á.L.P.-Q.); alexis.dereeper@ird.fr (A.D.); boris.szurek@ird.fr (B.S.); 2 Laboratorio de Interacciones Moleculares de Microorganismos Agrícolas, Departamento de Ciencias Básicas, Universidad de los Andes, Bogotá 111711, Colombia; d.osorio23@uniandes.edu.co; 3 Manihot Biotec, Departamento de Biología, Universidad Nacional de Colombia, Bogotá 111321, Colombia; remoram@unal.edu.co (R.E.M.); celopezc@unal.edu.co (C.E.L.); 4 Laboratorio de Micologíay Fitopatología de la Universidad de los Andes (LAMFU), Departamento de Ciencias Básicas, Universidad de los Andes, Bogotá 111711, Colombia; srestrep@uniandes.edu.co

**Keywords:** cassava bacterial blight, SWEET, host target genes, susceptibility, transcriptomics, EBE prediction

## Abstract

Transcription activator-like effectors (TALEs) play a significant role for pathogenesis in several xanthomonad pathosystems. *Xanthomonas phaseoli* pv. *manihotis* (*Xpm*), the causal agent of Cassava Bacterial Blight (CBB), uses TALEs to manipulate host metabolism. Information about *Xpm* TALEs and their target genes in cassava is scarce, but has been growing in the last few years. We aimed to characterize the TALE diversity in Colombian strains of *Xpm* and to screen for TALE-targeted gene candidates. We selected eighteen *Xpm* strains based on neutral genetic diversity at a country scale to depict the TALE diversity among isolates from cassava productive regions. RFLP analysis showed that *Xpm* strains carry TALomes with a bimodal size distribution, and affinity-based clustering of the sequenced TALEs condensed this variability mainly into five clusters. We report on the identification of 13 novel variants of TALEs in *Xpm*, as well as a functional variant with 22 repeats that activates the susceptibility gene *MeSWEET10a*, a previously reported target of TAL20*_Xam668_*. Transcriptomics and EBE prediction analyses resulted in the selection of several TALE-targeted candidate genes and two potential cases of functional convergence. This study provides new bases for assessing novel potential TALE targets in the *Xpm–*cassava interaction, which could be important factors that define the fate of the infection.

## 1. Introduction

Transcription activator-like effectors (TALEs) play a significant role for pathogenesis and virulence in several xanthomonads. Disruption or knocking out of some TALE encoding genes significantly affects the virulence of the pathogen or even abolishes pathogenicity [1,2,3,4,5,6,7,8,9,10,11,12]. This family of effectors shares a particular structure evolved to selectively bind plant host promoters and recruit the RNA polymerase complex in order to initiate transcription of downstream genes [13,14]. TALE proteins include a signal for translocation through the type III secretion system (T3SS) [15], nuclear localization signals (NLS) [16] and an acidic transcriptional activation domain (AAD) to promote transcription in the host cell [17]. The central region of the effector contains modular tandem repeats mainly composed of 33–35 amino acids [18,19] whose sequence varies primarily at residues 12th and 13th (so-called “repeat variable diresidue”, RVD). RVDs interact with DNA bases with a nucleotide recognition preference [20,21,22,23,24], ruling the DNA sequence recognition. Sequences targeted by TALEs are usually located within or near the promoter of target genes, and are termed Effector Binding Elements (EBEs) [1]. After secretion, TALEs interact with host transcription factors like TFIIAγ subunits, to induce polymerase II–dependent transcription [25].

Many host genes targeted by TALEs have been described for the rice-*Xanthomonas oryzae* pathovars *oryzae* (*Xoo*) and *oryzicola* (*Xoc*), but data are also available for wheat [26], citrus, cotton, pepper, tomato, and cassava-interacting *Xanthomonas* (reviewed by [4]). TALE targets whose activation during infection is promoting host disease are defined as susceptibility (*S*) genes, many of which are nutrient transporters. For example, TALE-mediated activation of SWEET sugar transporters in rice, cotton and cassava leads to promotion of virulence potentially through nutrient hijacking [10,27,28], while activation of sulfate transporter *OsSULTR3;6* in rice may alter redox status or osmotic equilibrium to interfere with defense signaling and induce water-soaking [3]. In wheat, induction by TAL8 of *Xanthomonas translucens* pv. *undulosa* of a gene involved in abscisic acid regulation alters water management in favor of the pathogen [26]. Other *S* genes include plant transcription factors, which control more complex host cellular processes through multiple indirect targets [2,6,9,10,12]. In pepper, AvrsBs3-dependent activation of *upa20*, a basic helix-loop-helix (bHLH) family member, induces hypertrophy [12], while AvrHah1-dependent activation of *bHLH3* and *bHLH6* upregulates cell wall-degrading enzymes [10]. In citrus, PthA4-mediated (and its functional equivalents) activation of the transcription factor *CsLOB1*, results in pustule development, which may be dependent on activation of DNA-interacting secondary targets [2,29].

*Xanthomonas phaseoli* pv. *manihotis* (*Xpm*), previously known as *Xanthomonas axonopodis* pv. *manihotis* [30], is a Gram-negative vascular pathogen responsible for Cassava Bacterial Blight (CBB). CBB has been reported in all the continents where cassava is grown [31]. As other xanthomonads, *Xpm* uses a suite of effectors to manipulate physiological processes in host plant cells. *Xpm* has up to 24 effectors that are translocated to the host cytoplasm through the T3SS (termed type III effectors), including members of the TALE family [32]. Population diversity studies using TALE1_Xam_ (a.k.a *pthB*) as a probe showed that all isolates from Latin-American and African *Xpm* collections carry TALEs [33,34,35,36,37]. Illumina-based genomic sequencing of more than 60 *Xpm* strains later confirmed that all strains contain at least one TALE [32,38]. Full *Xpm* TALE sequence is available for only eight effectors [8,39,40], and only two complete *Xpm* TALomes (whole set of TALEs present in a strain) have been reported so far [8,39].

Information about *Xpm* TALEs and their target genes in cassava has been growing in the last years. The first TALE characterized in *Xpm* is TALE1_Xam_ (contains 14 repeats), which has an important role in pathogenicity [40], but whose targets remain unknown [8,39,40,41]. Cohn and coworkers [8] deciphered the contribution of each of the five TALEs of strain Xam668, showing that mutant strains for TAL14_Xam668_ and TAL20_Xam668_ significantly affected pathogen fitness characterized by reduction of bacterial growth and/or symptom formation. Importantly they also demonstrated that TAL20_Xam668_ induces the *S* gene *MeSWEET10a*, which codes for a clade-III sugar transporter from the SWEET family.

Studying cassava pathogens is highly relevant since this crop is one of the most important starchy root crops for food security in America, Africa and Asia [42]. The annual worldwide production of cassava is estimated to 270 million tons and serves as a staple food for more than 800 million people, mainly in tropical countries. This and its tolerance to drought, make it an important food security crop [43,44]. CBB is the most devastating bacterial disease of cassava, causing losses that range from 12% to 100% [31] depending on environmental conditions [45,46], with incidences ranging between 25% and 100% [46,47]. CBB symptoms include angular leaf spotting, blight, wilt, gum exudation, vascular necrosis, stem canker and dieback (reviewed by [48]). In Colombia, CBB was first described in 1971 [35]. More recently, Trujillo and coworkers described the genetic structure of *Xpm* populations in the most prominent cassava production regions, showing high susceptibility of the most common cultivars grown and a complex pathogen population structure in some of these regions [49,50].

Science-based crop resistance improvement and disease tackling require a compilation of knowledge to understand the cellular and molecular bases of plant-pathogen interactions. As *Xanthomonas* pathogenicity notably relies on TALEs, knowledge on diversity and function of these effectors, in conjunction with resistance (R) proteins, are a cornerstone for resistance breeding in the affected crops [51,52,53,54]. Among the mechanisms of resistance associated with TALEs [55], recessive resistance is mediated by loss-of-susceptibility (LoS) alleles, where alternative alleles of an *S* gene that does not possess the EBE on the promoter sequence prevent the recognition and upregulation of the *S* gene (reviewed by [53]). As previously stated, only one *S* gene has been described for cassava (*MeSWEET10a*) as a direct target of TALE20_Xam668_ [8]. No LoS alleles, executor genes or TALE-recognizing nucleotide-binding leucine-rich repeat (NLR) receptors in cassava have been reported so far. In this work we aimed to characterize the TALE diversity of one of the best characterized *Xpm* populations in the world. We sequenced the repeat regions of 46 TALEs from 18 *Xpm* strains, including the complete TALomes from seven strains. We also aimed to determine functional diversity of the potential TALE-targeted genes and to propose candidates that could contribute to a better understanding of this pathosystem. The TALE-affinity analyses, host target prediction, and transcriptomics on cassava plants inoculated with the pathogen resulted in potential novel targets that might be relevant for pathogenesis. This study expands our understanding of the complex interactions between *Xpm* and cassava by picturing the TALE diversity in the context of this pathosystem.

## 2. Materials and Methods

### 2.1. Bacterial Strains, Plant Material, Culture Conditions and Media

All *Xanthomonas phaseoli* pv. *manihotis* strains were previously collected in diversity studies of this pathogen from our group (Laboratorio de Micología y Fitopatología de la Universidad de los Andes, Universidad de los Andes, Colombia). All the selected strains were pathogenic to the highly CBB-susceptible variety 60444. Isolates from two geographically distant cassava productive regions in Colombia were selected through maximization of differences of neutral genetic diversity. Additionally, two strains, one per region, isolated at least one decade before the collection dates of most isolates, were selected as reference strains. Bacteria were streaked on YPG (yeast extract 5 gL^−1^, peptone 5 gL^−1^, glucose 5 gL^−1^, agar-agar 15 gL^−1^) solid media [35] and incubated for 48 h at 28 °C or grown in Phi broth (yeast extract 1 gL^−1^, peptone 10 gL^−1^, casaminoacids 1 gL^−1^) [35] at 28 °C, under constant shaking at 220 rpm for 24 h. Cassava cuttings from the cultivar 60444 were planted on individual peat pots and grown under greenhouse conditions (27 °C ± 5 °C; photoperiod 12:12, relative humidity >60%). Bacterial inoculations were performed on 3-month old plants. In vitro-grown plants from the same cultivar were grown as described elsewhere [56].

### 2.2. Aggressiveness Assays and Bacterial Growth Curves

Bacterial aggressiveness was quantified as the leaf lesion area formed by *Xpm* upon inoculation of 3-month-old cassava plants. Bacteria were cultured in liquid media and washed with 10 mM MgCl_2_ sterile solution. Cell density was adjusted to an OD_600_ of 0.2 (c.a. 2 × 10^8^ cfu/mL). Adjusted bacterial suspensions were inoculated by placing a 10-µL drop of inoculum over a 2-mm (Ø) hole made with a cork borer through the leaf tissue. Plants were kept in a greenhouse under the same conditions described above. Lesions were individually photographed at 15 days post-inoculation (dpi) in a stereoscope and areas were measured using Image-J software (version 1.48) [57]. Each treatment was inoculated once in three individual plants, and the aggressiveness assay was replicated three times.

Bacterial growth in planta was assessed as described elsewhere [58] except that inoculations were performed on the variety 60444 and 0, 5, and 10 dpi were evaluated. Each treatment was inoculated once in three different plants and the bacterial growth assessment was replicated two times.

### 2.3. Genomic DNA Extraction, Restriction Fragment Length Polymorphism (RFLP), and Southern Blot Analysis

A single colony of each *Xpm* strain was grown on Phi broth overnight. DNA was extracted from 2-mL bacterial pellets using the Genelute^TM^ Bacterial Genomic DNA kit (Sigma-Aldrich, St. Louis, MO, USA) according to manufacturer’s instructions. Genomic DNA concentration was determined by gel quantification and spectrophotometry on a NanoDrop^TM^ 1000 (Thermo Fisher Scientific, Waltham, MA, USA). Ten micrograms of each genomic DNA were digested with 120 units of *Bam*HI-HF (NEB, Ipswich, MA, USA) sequentially added (3 times 40 units) over a 24-h incubation period. *Bam*HI-digested DNA was precipitated using sodium acetate and ethanol [59] and then resuspended in TE solution. Electrophoresis of digested DNA was performed on a 0.8% agarose gel on 0.5× TBE (Thermo Fisher Scientific, Waltham, MA, USA); migration was performed at a constant power of 5 Watts for 20 h, in a cold room and with periodic buffer renewal. DNA blotting was performed according to Roche’s instruction manual for Digoxigenin application to filter hybridization [60]. The probe was synthesized from a conserved portion of the TALE1_Xam_ (from nucleotide 2909 to 3409) DNA contained in pF3, a pBluescript derivative with a 5.4-kb fragment from CFBP1851 plasmid p44 containing the full TALE1_Xam_ gene [35]. The probe was labeled with digoxigenin by means of the Random Primed DNA Labeling kit (Roche Diagnostics GmbH, Mannheim, Germany). Luminescent detection was achieved using chloro-5-substituted adamantyl-1, 2-dioxetane phosphate (CSPD) according to manufacturer’s instructions. Chemiluminescent images were acquired on the ChemiDoc XRS + System (BioRad, Hercules, CA, USA) with the Chemi Hi Resolution protocol and signal accumulation mode.

### 2.4. Isolation and Sequencing of TALEs

The isolation of TALE genes was achieved by two different approaches. The first one consisted of PCR amplification of the central repeat region using GoTaq polymerase (Promega, Madison, WI, USA) and subsequent cloning into pGEM^®^-T easy (Promega, Madison, WI, USA). Amplification was performed using 200 nM of primers 427 Fw (5′-CGGTGGAGGCAGTGCATG-3′) and 428 Rv (5′-ATCAGGGCGAGATAACTGGGC-3′); 1× GoTaq Green buffer, 1.5 mM MgCl2; 100 µM dNTPs, 20 mM betaine, 0.025 GoTaq polymerase units/µL and a total amount of 40 ng of template DNA in a final volume of 20 µL. PCR conditions were as follows: Initial denaturation at 94 °C for 3 min; then 25 cycles of 94 °C for 40 s, 60 °C for 40 s, and 72 °C for 3 min and 30 s. Cloning into pGEM^®^-T easy was performed according to manufacturer’s instructions. Ligation products were electroporated into E. coli DH5α cells. Screen of clones harboring inserts was performed using blue/white screening on LB agar using 5-bromo-4-chloro-3-indolyl-β-D-galactopyranoside (X-gal) and a PCR-based confirmation (as described above).

The second approach was based on direct cloning of TALE genes into pBlueScript. RFLP data was used to calculate the position of each TALE in the electrophoresed *Bam*HI-digested DNA. For each strain, 70 µg of DNA were digested. Electrophoresis was carried out in a 0.8% agarose gel on 1× TAE; migration was performed at a constant power of 5 Watts for 5 h at room temperature. Gels were stained using a 3× GelRed (Biotium, Fremont, CA, USA) pool. Gels were quickly visualized in a ChemiDoc XRS+ System (BioRad, Hercules, CA, USA), regions where the expected TALE bands were excised and recovered using the Zymoclean™ Gel DNA Recovery Kit (ZymoResearch, Irvine, CA, USA) according to manufacturer’s instructions. pBlueScript was digested with *Bam*HI-HF (NEB, Ipswich, MA, USA) and dephosphorylated with Antarctic Phosphatase (NEB, Ipswich, MA, USA) according to manufacturer’s instructions. Eluted DNA was ligated with the prepared vector with T4 DNA ligase (Thermo Fisher Scientific, Waltham, MA, USA) at 22 °C overnight. Transformation of ligations, screening and confirmation were performed as described earlier.

Sequencing of isolated TALE genes was carried out by standard Sanger chemistry (Macrogen Inc., Seoul, Korea) and the assembly of reported fragments was performed using the Geneious software (version R11). In the case of TALE genes obtained by PCR, they were only considered accurate if they were found in at least two rounds of independent amplifications and cloning, or if they were also cloned directly from digested DNA.

### 2.5. TALE Characterization and Clustering

Assembled TALE sequences were treated and classified in variants using an in-house script on R (version 3.6.1). TALEs were clustered by predicted DNA binding specificity using FuncTAL software (version 1.1) [61]; default parameters were used. TALEs were also clustered by nucleotide repeat composition using DisTAL software (version 1.1) [61]; default parameters were used.

### 2.6. In-Vitro Plant Inoculation and Cassava RNA Extraction

*Xpm* strains were cultured in liquid media and prepared, as described earlier. Inocula were adjusted to an OD_600_ of 0.02 (ca. 2 × 10^7^ cfu/mL) in a 10-mM MgCl_2_ solution. In-vitro propagated plants were inoculated with bacterial suspensions or mock solution (10-mM MgCl_2_) with a swab on axial and abaxial surfaces of punctured leaves (nine needle punctures per leaf); each treatment was inoculated on three leaves per plant and on three different plants. Tissue surrounding inoculated punctures was collected at 50 hpi using a 3-mm diameter cork borer. Total RNA was extracted with the Invitrap Spin plant RNA minikit (STRATEC, Birkenfeld, Germany), using the RP buffer per manufacturer’s instructions. Total RNAs were treated with RNase-free DNase I (Thermo Fisher Scientific, Waltham, MA, USA) and quality was controlled with the RNA 6000 Nano Kit (Agilent, Santa Clara, CA, USA).

### 2.7. RNA Sequencing and Transcriptome Analysis

Barcoded, paired-end (150-bp inserts) libraries were constructed with the TruSeq RNA Library Prep Kit (Illumina, Vancouver, British Columbia, Canada), per manufacturer’s instructions. RNA libraries were pooled and sequenced on four flow cells of the NextSeq500 System. RNAseq analyses were performed using the Kallisto pseudo-mapper [62] and EdgeR [63] for differential expression analysis by comparing against the mock-inoculated treatment on R (version 3.6.1). GO-term enrichment analyses were performed using the topGO package [64].

### 2.8. TALE Target Prediction and Candidate Analysis

TALE targets were predicted using four different software: TALVEZ [65], TAL Effector Nucleotide Targeter 2.0 (TALENT 2.0) [66], TALgetterLong [67] and PrediTALE [68]. The *Manihot esculenta* promoterome (1-kb sequences preceding annotated translational start sites) was extracted from Phytozome’s cassava genome version 6.1 [69], by means of the Biomart tool, and it was used as input for target prediction. All the algorithms were run using the default parameters. Output data were merged and compared on R (version 3.6.1) using an in-house script.

### 2.9. Semi-Quantitative and Quantitative RT-PCR

*Xpm* strains were cultured in liquid media and prepared as described earlier. Bacterial inoculum was adjusted to an OD_600_ of 0.5 (c.a. 5 × 10^8^ ufc/mL) in a 10-mM MgCl_2_ solution. Bacterial suspensions or the mock solution (10-mM MgCl_2_) were infiltrated into leaves of 3-month old adult plants grown from stakes, by means of a needleless syringe; each treatment was inoculated on one leaflet per plant and on three different individuals. Infiltrated tissue was collected at 50 hpi in sterile tubes containing RNA-free glass beads, samples were frozen with liquid nitrogen and ground by vortexing. Total RNA was extracted as described earlier. cDNA synthesis was performed with the High-Capacity cDNA Reverse Transcription Kit (Applied Biosystems, Walthman, MA, USA), per manufacturer’s instructions. Semi-quantitative RT-PCRs were performed with a 20-µL reaction mix per tube containing 1× of 5× GoTaq Green Buffer, 1.5 mM of MgCl_2_, 100 µM of a dNTP mix, 0.2 µM of each primer, 5 ng/µL of cDNA, and 0.025 U/µL of GoTaq polymerase (Promega, Madison, WI, USA). Amplification was programmed as follows: one step at 95 °C for 3 min, followed by a variable number of cycles (20 to 28, as needed) of 95 °C for 30 s, 60 °C for 40 s, and 72 °C for 30 s. Amplicons were resolved in 1% agarose gels on 0.5× TBE. RT-qPCRs were performed on a 7500 Fast & 7500 Real-Time PCR System. Each well contained 10 µL of the following reaction mix: 1× of 2× SsoFastTM EvaGreen Supermix with Low ROX (BioRad, Hercules, CA USA), 300 nM of each primer, and 20 ng/µL of cDNA. PCR cycling was as follows: one step at 95 °C for 30 s, followed by 40 cycles of 95 °C for 5 s, and 60 °C for 30 s; data was acquired during the second step of each cycle. The melting curve was evaluated from 65 °C to 95 °C. Primers for reference and candidate genes are summarized in Table 1.

### 2.10. Statistical Analysis and Packages

All the statistical analyses were performed in R (version 3.6.1). Aggressiveness data was analyzed using an in-house script through a linear mixed model—packages lme4 [59] and multcomp [60]- for log-transformed data, where strain was used as predictor variable with fixed effects, and replicates in time were included as the random effect. All the statistical comparisons were performed at a 0.95 significance level. Correlation analyses were performed by using ggpubr [70]. RT-qPCR data were analyzed using pcr package [71], where a two-tailed *t*-test (alpha 0.05) was applied to the normalized expression values (normalized by using tubulin gene as reference) of each target gene in the inoculated treatment versus the mock-inoculated treatment. Boxplots, barplots, dotplots, and heatmaps were created with ggplot2 [72], gplots [73] package. EBEs were depicted in the promoter contexts by using the gggenes package [74] or Geneious software (version R11). Distance-based trees were modified on Geneious software (version R11).

## 3. Results

### 3.1. Aggressiveness of Selected Xpm Strains Is Homogeneous on Cassava Cultivar 60444

*Xpm* Colombian strains were selected based on three criteria: year of collection, the edaphoclimatic zone (ECZ) of CBB-endemic cassava-growing lands [35], and previous AFLP- and MLVA-based diversity information [49,50]. From the two geographically distant ECZs, which harbor genetically differentiated *Xpm* populations [50], a total of 18 strains representative of the Colombian *Xpm* diversity were selected (Table 2 and Supplementary Figure S1). It is also worth noting that strains UA318 and UA681 were previously used in mapping studies for QTL of resistance in cassava against CBB [75]. Also, a subset of them was sequenced by Illumina (strains CIO151, CFBP1851, UA226, and UA306) [32,38], and/or pathotyped on a set of cassava varieties (strains UA226, UA306, UA318, UA531 and UA681) [50].

**Figure 1:**
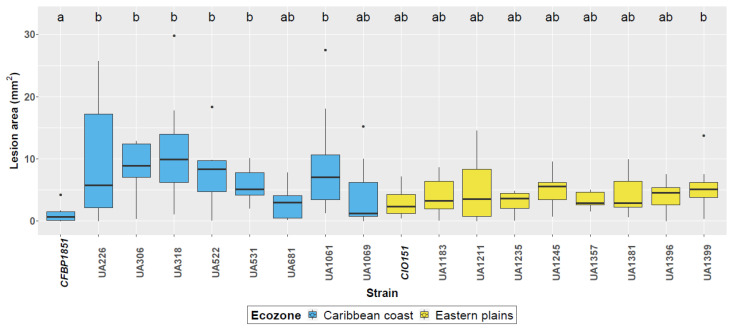
Aggressiveness of selected *Xpm* strains. Lesion areas in squared millimeters are displayed for each strain. Strains used as reference for each region (CFBP1851 and CIO151) are bolded and italicized in the x-axis. Boxes for strains isolated in the Caribbean coast region are colored in blue, while boxes for strains isolated in the Eastern plains are colored in dark yellow. Boxplots were constructed with measurements from three biological replicates with three replicates each. A linear mixed model was fitted to the log10 transformed data, with replicates of the experiment as random effect, which showed to be near to zero. Different letters indicate significant differences according to a post-hoc Tukey’s test (α = 0.05).

Aggressiveness of the strains was evaluated based on leaf lesion area on cassava adult plants from the cultivar 60444 at 15 dpi (Figure 1). All the strains were pathogenic and caused symptomatic infections, with water-soaked angular lesions and necrosis in some parts of the tissues. The biological variation among treatments was considerably high. However, lesion areas caused by isolates from the Eastern plain region show narrower inter-quartile ranges (IQRs) for most of the treatments when compared to Caribbean coast strains, which would point to a lower variability in aggressiveness for these strains. The reference strain from the Caribbean coast, CFBP1851, consistently caused the smallest lesions on 60444, while largest lesions were observed with strains UA318 and UA1061. However, due to the considerable variance, little could be inferred from this analysis, except that *Xpm* aggressiveness on 60444 seems to achieve homogeneous levels after 15 dpi and cannot be correlated with the AFLP- and VNTR-based genetic neutral diversity used as a criterion to select strains.

### 3.2. RFLP Analysis Highlights TALome Patterns with Two to Six TALEs Per Strain and Restricted Size Ranges

**Figure 2:**
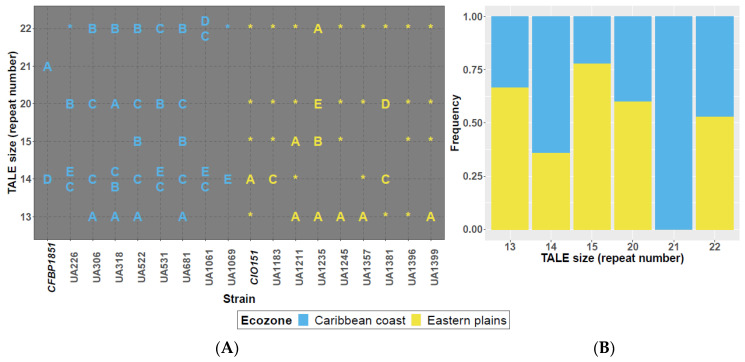
RFLP analysis, sequenced TALE gene variants and distribution. (**A**) TALome representation based on RFLP analysis and sequencing. Each band observed in the RFLP blot indicates the presence of at least one TALE gene. Cloned and sequenced TALE gene variants were first classified according to their size in terms of repeat number and then a letter was assigned to each variant according to the translated RVD sequence. Different letters in a row indicate different RVD strings for each TALE size. TALE genes that were only detected based on the RFLP analysis are indicated with a star. Reference strains of each ecozone are shown bolded and italicized in the *x*-axis. (**B**) TALE size prevalence according to regions. Frequencies are based on the size and number of bands observed on the RFLP analysis.

RFLP analysis was performed using a 504-bp probe corresponding to a segment of the C-terminal region of the TALE1_Xam_ gene (a.k.a *pthB*). Figure 2A shows TALome patterns, where each band observed in the RFLP blot is represented by a letter or a star. Among the 18 tested strains, TALomes are consistently composed by effectors with 13, 14, 15, 20 or 22 apparent repeats, while bands corresponding to effectors sizes ranging from 16 to 19 repeats are completely absent (bimodal size distribution). RFLP analysis was followed by cloning and sequencing of most of the TALE genes present in the studied *Xpm* strains. From the 73 bands observed in the blot (Supplementary Figure S2), we were able to sequence 46 genes. TALE genes will be named according to their predicted number of repeats (e.g., TALE20 has 19.5 repeats). Variants are indicated in Figure 2A through letter codes according to RVD sequence classes of TALEs with the same repeat number. Gene names included the suffix *Xpm*. However, for the sake of readability of this document, suffix will be obviated. Eighteen RVD-based variants were found.

As shown in Figure 2, eighteen different TALE variants were detected in the Colombian strains. Thirteen of them are novel variants, whereas five are variants that were previously reported in *Xpm* (namely, TALE14C, TALE14D, TALE15B, and TALE22A [32,40]). Variants TALE21A, TALE14A, and TALE14D were only observed in the reference strains, collected in the 90s, a possible indication of TALE selection through time. TALE22 variants are the most commonly distributed (present in 17 out of 18 strains), followed by TALE20 and TALE14 variants (15 and 14 out of 18 strains, respectively). Interestingly, distribution of sizes according to the source edaphoclimatic zone (Figure 2A,B) indicates a bias on the TALome content for TALE13, TALE14, and TALE15 variants. TALE14 variants are more widespread among isolates from the Caribbean coast, while TALE13 and TALE15 variants are more frequently found among strains from Eastern plains. A statistically relevant correlation between the absence of TALE14 and the presence of TALE15 variants was found by Spearman correlation test (Rho = −0.535, *p*-value = 0.022), but it was not significant for TALE13 vs. TALE14 variants (Rho = −0.378, *p*-value = 0.122). Correlations between TALE presence and aggressiveness data were also tested, but no significant associations were found (data not shown). At RVD sequence level, TALE13 has a unique widespread variant, while TALE15 shows two variants differentiated by only one RVD (change of the nucleotides that code for a NN to a NS in the RVD of the twelfth repeat; NN12NS). TALE14, TALE20, and TALE22 showed at least four variants each by the coding sequence of at least one RVD and a maximum of 11, 6, and 13 RVDs, respectively. Table 3 details the RVD sequences of sequenced TALE variants.

### 3.3. Five TALE Clusters with Similar Predicted DNA-Binding Affinities

**Figure 3:**
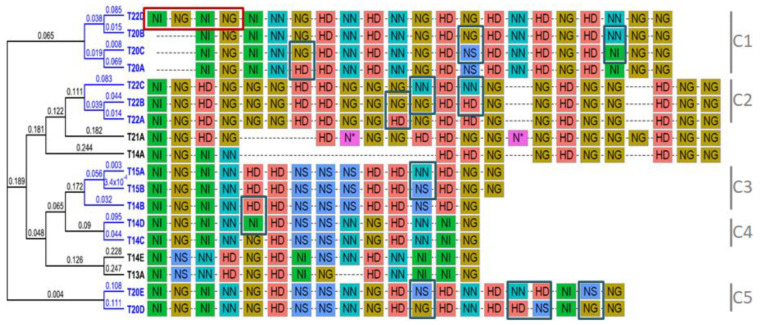
RVD sequence clustering based on predicted DNA-binding affinity. FuncTAL was used to estimate distances based on the predicted TALE affinity. The dendrogram on the left shows the estimated relationship, where numbers represent the distance between the position weight matrices calculated for each TALE. In branch labels, T stands for TALE. Blue branches limit each cluster, and black terminal branches indicate that the corresponding effector was not clustered (orphans). Central alignments depict RVD strings for each TALE variant, where each box represents a repeat with its RVD. Boxes are colored according to RVD type. Dotted lines show gaps inserted by the alignment procedure. Labels on the right indicate tags of clusters as defined by blue branches. The red square shows a possible duplication of the first two repeats, blue squares show repeat swaps between TALE variants.

To investigate potential DNA-binding affinities and redundant functions for TALE variants found among these isolates, we used the program FuncTAL. FuncTAL clusters TALEs based on correlations between potential target DNA sequences predicted from each RVD string [61]. Figure 3 shows the dendrogram that resulted from the analysis of the 18 TALE variants reported in Table 3. The color code used to represent RVD types in the analyzed TALEs highlights interesting general features: (i) the first repeat invariably contains NI as RVD, and most of the effectors initiate the repeat sequence with a NI-NG-NI-NN repeat block; (ii) the last half-repeat is consistently NG, and the repeat region of the majority of variants ends with an NG-NG repeat block; (iii) NG is the most frequent RVD in *Xpm* TALEs while N* is only present in one effector extracted from reference strain CFBP1851, and (iv) repeats with NS tend to be generally restricted to central positions along the repeat array.

Blue terminal branches on the dendrogram highlight five binding affinity clusters (a maximum of 3 individual RVD mismatches between each pair of compared RVD strings), which cover most of the variants for TALE15, TALE20 and TALE22 (Figure 3). In general, TALE variants in the same affinity group have the same number of RVDs. However, two out of the five clusters include variants with different number of repeats, and the alignment of RVDs shows that this is due to duplication/deletion of whole repeats at the beginning or end of the repeat region. Among them, TALE22D variant shares the whole RVD sequence with TALE20B variant, which indicates that this novel TALE might also promote transcription of the S gene *MeSWEET10a*. It is also worth noting that variants TALE20D and TALE20E exhibit a considerable number of differences relative to A, B and C variants, and instead show similarities to TALE15 and TALE14 variants. On the other hand, black terminal branches point to TALE variants whose predicted affinities are considerably different to the rest and cannot be grouped, suggesting that they do not share EBEs with TALEs from any of the clusters.

In parallel, a repeat-based phylogeny was constructed using the software DisTAL [61] (Supplementary Figure S3). Results show nearly the same clustering patterns as observed with FuncTAL, but TALE20, TALE21, and TALE22 variants are grouped into one big clade, while TALE15 and TALE14 variants (except for TALE14E) constitute another big clade. The nucleotide versions of TALE13A and TALE14E variants form a big clade that seem to be at the origin of the two above-mentioned clusters.

### 3.4. Expression Profiles of Plants Inoculated with UA681 and UA1061 Are Highly Similar

**Figure 4:**
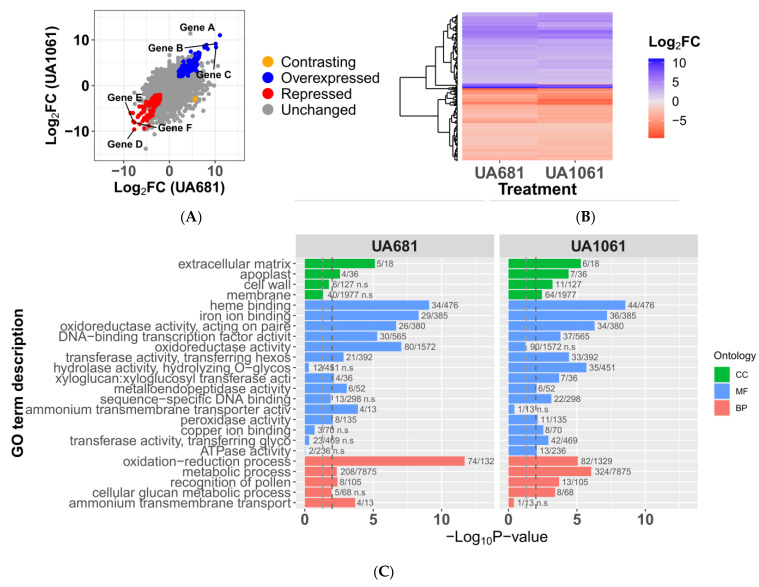
Overview of RNAseq data and GO-term enrichment for differentially expressed genes. (**A**) Comparison of cassava gene expression profiles observed at 50 hpi of 60444 plants inoculated with *Xpm* strains UA681 and UA1061; mock-inoculated plants were used to determine the differentially expressed genes in each case. Plot shows the shared differentially upregulated genes (DUGs) in blue and differentially down-regulated genes (DDGs) in red, while a gene that has a contrasting transcriptional behavior between the two bacterial treatments is marked in yellow. Marked genes correspond to the three shared DUGs and DDGs with most extreme response magnitudes: A: *Manes.06G123400*; B: *Manes.17G063800*; C: *Manes.07G120000*; D: *Manes.17G042600*; E: *Manes.05G151100*; F: *Manes.18G029800*. (**B**) Heatmap of the shared DUGs and DDGs transcriptional behavior. (**C**) GO-term enrichment analysis showing the cellular component (CC), molecular function (MF) and biological process (BP) categories that are significantly enriched in the differentially expressed genes in UA681- or UA1061-treated plants. The corresponding GO term IDs are summarized in the Supplementary Table S3. Discontinuous lines show the *p*-value thresholds corresponding to 0.05 (blue) and 0.01 (dark gray).

To gain insights into the cassava genes targeted by the TALomes of Colombian *Xpm*, we performed RNA-seq experiments of cassava leaves challenged with strains UA681 and UA1061. These strains were selected because their TALomes are fully sequenced (Figure 2), and they collectively contain eight different TALE variants, allowing to cover four out of the five binding affinity clusters (see Figure 3), as well as the “orphan” variants TALE13A and TALE14E. Moreover, strain UA1061 lacks a TALE20 variant but it is as aggressive as others that contain TALE20, therefore, we aimed at investigating alternative explanations for this aggressiveness that could provide insight into the pathogenesis of *Xpm*. To this end, a mock solution (10-mM MgCl_2_) or *Xpm* strains were inoculated into two-month old cassava plants propagated in-vitro, and tissue was harvested at 50 hpi. As illustrated in Figure 4A, expression profiles are remarkably similar and some of the most up- (Log_2_FoldChange ≥ 2 and *p*-value < 0.001) and down-regulated (Log_2_FoldChange ≤ −2 and *p*-value < 0.001) genes are shared by both treatments (upregulated genes *Manes.06G123400*, *Manes.17G063800*, *Manes.07G120000*; downregulated genes *Manes.17G042600*, *Manes.05G151100*, *Manes.18G029800*). However, treatment with UA681 significantly affected the transcription level of 588 genes, while UA1061 significantly affected that of 1021 genes. When analyzed as a whole, most of the differentially expressed genes (DEGs) shared by both treatments show the same transcriptomic profile (271 out of 273 genes, Figure 4B). The top three upregulated DEGs common to both treatments are *Manes.06G123400*, *Manes.17G063800* and *Manes.07G120000* which respectively encode the *S* gene *MeSWEET10a*, an oxidoreductase 2OG-Fe II oxygenase family protein, and a wall-associated receptor kinase galacturonan-binding. The top three common downregulated DEGs encode a tyrosinase (*Manes.17G042600*), a cytochrome P450 71B21-related protein (*Manes.05G151100*) and a protein that belongs to the mitochondrial calcium uniporter family (*Manes.18G029800*). The Gene Ontology (GO) term enrichment analysis (Fisher’s test, α = 0.01) performed on shared DEGs (Figure 4C) shows that, as defined by cell compartment, most of the differential transcriptional activity accounts for proteins that localize to the cell membrane or the apoplast. Moreover, the molecular function and biological process classifications point towards a response that involves a significant number of genes altering cell redox status, glucan metabolism, and nitrogen transport.

### 3.5. Potential TALE Targets Are Involved in the Manipulation of Host Cell Redox Status and Nutrient Transport

Correlation of inoculated host gene expression profiles with EBE prediction is a powerful approach to find genes targeted by TALEs. The promoterome (defined as the 1-kb regions that flank annotated translational start sites) of cassava inbred line AM560-2 [76] was used as input to predict the EBEs of each of the 18 TALE variants discovered in this study. We used four different on-line available software: TALVEZ [77], TAL Effector Nucleotide Targeter 2.0 (TALENT) [78], TALgetter [65], and PrediTALE [66]. The output of each program was restricted to the first 400 results and the rank of each predicted EBE was used as a homogenized score to enable comparisons. We cross-referenced the DUGs with the set of predictions for the TALomes of each inoculated strain. Results for UA1061 show that from the 320 DUGs, only 36 were predicted to have at least one EBE on the promoter region, while for UA681 the ratio was 69 out of 396.

**Figure 5:**
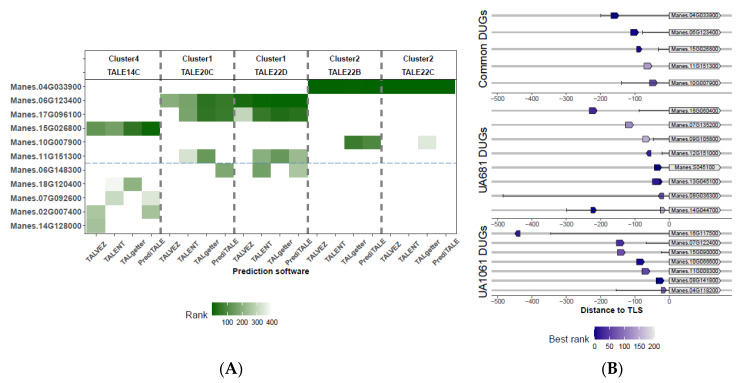
Differentially upregulated genes with predicted EBEs and their promoter context. (**A**) Shared DUGs with EBE predictions for TALE variants or TALE clusters shared by both strains. The heatmap shows the best candidates and the EBE-prediction quality from each software. Top labels indicate the TALE variant predicted to bind each gene and the DNA-binding affinity cluster where it belongs. Rank indicates the position of the prediction (based on the score) among the set of the top 400 predictions. Genes over the dashed line are considered as the best optioned candidates (see text). (**B**) Promoter context and predicted EBE position for candidates retained after the second filter (see text above). Genes are represented by gray arrows, while EBEs are represented by short arrows with a color code according to prediction quality. Direction of short arrows indicates if EBEs are located in the plus (pointing to right) or the minus (pointing to left) strands. Transcription start sites (as annotated on Phytozome’s cassava genome v6.1) are marked as vertical black lines connected to the translational start site with a solid black line to represent the 5′-UTR.

The study of common DEGs could shed light on potential hubs used by the pathogen to manipulate the host. When predictions for the two TALomes and transcriptomics from plants inoculated with both strains were compared, we detected six candidate genes (Figure 5A, first six genes from the top to the bottom of the figure) with high-quality EBE predictions whose transcription could be upregulated by TALEs from clusters 1, 2, and 4 (see threshold in Figure 5A). Table 4 details the annotation and the prediction quality obtained for the proposed candidates. In the analysis of individual TALomes (Supplementary Figure S4), a clear signature prediction/expression for probable candidates was observed for 19 additional genes in the case of UA681, and 7 additional genes for UA1061 (see Table 4). Distance to the translation start site and orientation of EBEs are two factors known to affect transcriptional activation rate driven by TALEs [3,67]. Cernadas and coworkers [3] found that EBEs in real targets ranked consistently on the first 200 predictions, and their distance to annotated transcriptional start site (TSS) generally ranged between 152 bp upstream and 63 bp downstream; being this latter parameter the most predictive descriptor. We applied a second filter by using information of EBE rank among predictors (best prediction) and distance to TSS (when available) and translational start site (TLS) to further classify potential real EBEs. Figure 5B shows EBE predictions in the promoter context of the retained candidates. Twenty candidate genes (see Table 4) out of the 34 and their predicted EBEs fell into the predictor ranges, and only three of them are located on the minus strand (reverse orientation). Among these candidates, we found potential novel targets that were not found in previous studies [8,39,41] and could be directly linked to defense regulation, like two different Abscisic Acid Receptor PYL4 encoding genes (*Manes.10G007900*, *Manes.07G135200*), a Rho GTPase-Activating Protein Ren1 encoding gene (*Manes.18G060400*), and an ammonium transporter (*Manes.08G141800*) among others.

Since predictions were based on the well-annotated cassava genome from inbred line AM560-2, we blasted the candidate genes against the recently published genome from variety 60444 [68] to extract the corresponding promoters and check for EBE presence. Among the 34 candidates, exact EBE matches were found for 29 of them, and imperfect matches (from 1 to 3 mismatches) for three more genes. Predicted EBEs for *Manes.S013800* (coding for a peptidase of plants and bacteria) and *Manes.04G096600* (coding for a Xyloglucan:Xyloglucosyl Transferase) were not found in the 60444 promoters. All the EBE predictions, except for two cases (*Manes.09G134100* and *Manes.04G109300*), were found in similar positions relative to TLS, with a maximum shift of 30 bp (Supplementary Table S1).

To explore the effect of potential candidates on transcriptome modification from a functional point of view, a GO term enrichment analysis for each transcriptome was run keeping only DUGs that contained EBEs for the corresponding TALome (Supplementary Figure S5). For strain UA681, there is a significant (Fisher’s test, α = 0.01) term enrichment mainly for molecular functions and biological processes related to oxidoreductase activity, indicating that TALEs might be involved in host cell redox status manipulation. However, the analysis does not show the same landscape for plants inoculated with UA1061, where none of the categories (cell compartment, molecular function, and biological process) seemed to be significantly enriched. Nevertheless, the ammonium transport is shown as a differential characteristic possibly linked to TALE activity from UA1061 TALome.

### 3.6. Candidate Gene Expression Profiles Are in Agreement with Trends Observed in RNAseq and Previous Reports

**Figure 6:**
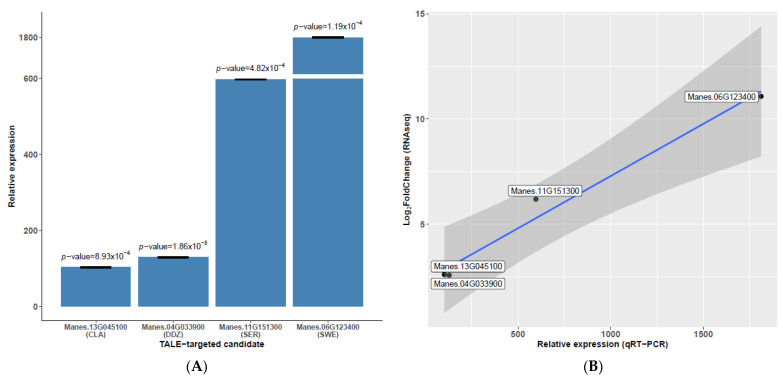
RT-qPCR validation of expression profile for selected genes. (**A**) Relative expression of four DUGs in UA681-inoculated vs. mock-inoculated plants. Main bars show the average of three independent replicates, and error bars show standard deviation. Note that *y*-axis has been broken to allow visualization of less expressed genes. *p*-values correspond to a two-tailed *t*-test (α = 0.05) comparing normalized expression in the UA681-inoculated and mock-inoculated treatments. X axis shows gene identifiers and a three-letter code to denote each gene. (**B**) Relative expression tested by RT-qPCR plotted against RNAseq profiles for the selected candidates. White boxes show the gene identifiers.

We confirmed the expression profile of some candidates through RT-qPCR with RNA extracted (at 50 hpi) from adult plants inoculated with UA681. The candidate genes were *Manes.13G045100*, *Manes.04G033900*, *Manes.11G151300*, *Manes.06G123400*. The four candidates have strong predictions; but first two genes have fold-changes near 2 (threshold to define upregulated genes), while the latter two have large fold-changes (6.19 and 11.06, respectively). Relative overexpression is statistically significant (Figure 6A), and expression patterns are well correlated between RNAseq data and RT-qPCR data (Figure 6B). Cohn and colleagues [8,39] demonstrated the TALE-dependent overexpression of 28 cassava genes in the Xam668-cassava infection context. RVD sequences of the TALEs of the UA681 strain are very similar to the ones present in Xam668: TAL13, TAL14 and TAL15 have the same RVD sequence, while TAL20 differs in 4 central RVDs and TAL22 shows the HD11NG change. Cross-reference of validated targets with the set of common and UA681 DUGs shows that 19 of these validated candidates are present in this set of transcriptionally activated genes, but only seven of them had predicted EBEs. Previously unreported candidates found in this study might be specifical targets of TAL20C and TAL22B variants. The last column of Table 4 shows the validated candidates and which TALE is potentially responsible for their transcriptional activation.

### 3.7. Potential Functional Convergence within TALome Members and Among Expanded TALome

**Figure 7:**
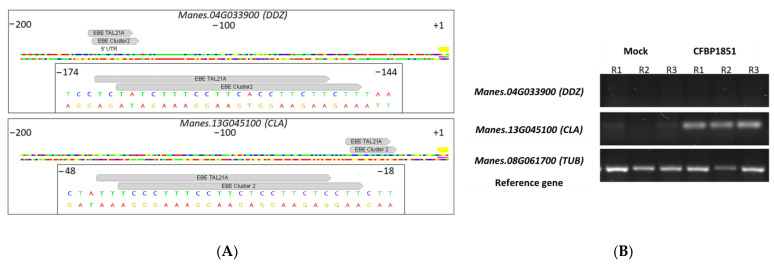
EBE predictions and semiquantitative RT-PCR in the context of a convergent activation of two candidates by two different TALEs. (**A**) Overlapping EBEs for TALE21A and TALE variants of the cluster 2 in the context of promoters for *Manes.04G033900* and *Manes.13G045100* genes. Each case is displayed as an overview and a zoom on the region of interest. EBEs are shown as gray arrows pointing to the right. CDS is represented as a yellow band, while 5-UTR region (only annotated for *Manes.04G033900*) is shown as a gray band. TSS or TSL was centered on the nucleotide number 1000, so coordinates are a reflect of this setting. (**B**) Semiquantitative RT-PCR for genes *Manes.04G033900* and *Manes.13G045100* on cDNA from cassava leaves inoculated with CFBP1851 or with a 10-mM MgCl_2_ solution (Mock). R1, R2 and R3 are independent replicates of the inoculation. Tubuline (TUB—*Manes.08G061700*) was used as the reference gene for normalization.

Functional convergence among TALEs is defined as two or more distantly related TALEs targeting the same gene, using the same, overlapped or completely different EBEs. This feature can be assessed at two different levels: (i) intra-TALome, for genes that are targeted by at least two TALEs expressed by the same bacterium (functional redundancy) and (ii) among the TALE variants present in the bacterial population. We did not find any cases of intra-TALome functional convergence. However, analyses extended to all the TALEs isolated in this study allowed us to track potential cases of functional convergence. Due to marked similarities between the affinity of clusters 1 and 5 (both harboring TALE20 variants), these convergence analyses did not take them as individual clusters. This analysis considered only DUGs (from UA681 or UA1061 treatments) with EBE for two or more TALEs from different clusters and resulted in eight candidates (Supplementary Table S2). The gene *Manes.04G033900*, which encodes a Dof Domain, Zinc Finger Protein, showed the strongest evidence for convergence. This gene was upregulated in both treatments and has been already validated as an actual target for TALE22_Xam668_ [8]. Theoretically, this gene is targeted by TALEs from cluster 2 (TALE22A, TALE22B and TALE22C) and TALE21A. Likewise, a second validated target for TALE22_Xam668_, upregulated in both RNAseq treatments and with EBE predictions for TALE21A, *Manes.13G045100* (coding for a Clavata3/ESR (CLE)-Related Protein), was found among the potential candidates. EBE predictions for TALE21A are almost totally overlapped with EBE predictions for cluster 2, sharing 20 base pairs in both cases (Figure 7A). Since predictions are well-supported and these genes were already validated as real TALE22 targets, we decided to test activation of these genes by inoculating cassava leaves with strain CFBP1851, a strain that lacks TALE22 variants but contains TALE21A. RNA was extracted after 72 hpi (since aggressiveness of this strain is considerably low. See Figure 1) and semiquantitative RT-PCR was performed on the derived cDNA. Figure 7B shows results for both genes. Strain CFBP1851 was able to differentially upregulate gene *Manes.13G045100* when compared to the mock-inoculated treatment, indicating that this is probably a case of TALE convergence (still, activation by a non-TALE factor cannot be ruled out with this experiment). However, transcripts from gene *Manes.04G033900* were not detected by the RT-PCR in plants treated with CFBP1851, indicating that TALEs from this strain are not able to activate this target.

## 4. Discussion

This study started with a selection of *Xpm* strains based on neutral genetic diversity at a country scale that allowed us to depict the TALE variation among strains isolated from different cassava productive regions over different years in Colombia. Southern blot analysis showed that *Xpm* strains carry TALomes with a bimodal size distribution, and sequence analysis showed thirteen novel TALE variants. Sequence clustering by predicted DNA-binding affinity allowed us to condense this variability into five clusters and four unrelated TALE variants. A complementary transcriptomic approach allowed us to move from diversity to function and to screen host genes that might act as CBB susceptibility determinants. Transcriptomics showed that redox processes and nutrient transport are upregulated during infection, and some of the involved genes are potentially targeted by TALEs. Importantly, our results also suggest that TALEs different from TALE20 might activate the transcription of MeSWEET10a and potentially confer aggressiveness to those strains lacking TALE20 in *Xpm* populations. This study sets new bases for assessing unidentified TALE targets in the *Xpm*-cassava interaction, which could be important factors that define the fate of the infection.

TALEs play major roles in the pathogenesis of several xanthomonads (reviewed by [4]), mainly by conferring bacteria the ability to create a favorable niche in the plant vascular system. In some cases, *Xanthomonas* spp. pathogenicity is drastically altered by the activity of a given TALE [69,79]. Bart and coworkers [32] found a positive trend between the number of TALEs per TALome vs. aggressiveness and virulence assessed in 18 strains of *Xpm*. In our study, we profiled the aggressiveness of 18 *Xpm* strains and found that only CFBP1851 shows reduced capacity to cause lesions. Interestingly, this strain does not carry TALE20 or any TALE20 variant that could activate the *S* gene *MeSWEET10a* [8], which could explain reduced aggressiveness. Strains UA1061 and UA1069 also lack this TALE variant, but their aggressiveness is average. However, UA1061 possess two variants of TALE22, one of them with an RVD sequence similar to that of the TALE20B variant plus a duplication of the first two repeats (Figure 2 and Figure 3). This new TALE22 variant (TALE22D) could explain the fact that strain UA1061, and potentially UA1069, do not have a decrease in aggressiveness even if they lack TALE20 variants. The DisTAL analysis (Supplementary Figure S3) indicates that TALE22D shares ancestry with TALE20B, and, based on the transcriptomic profile of UA1069-inoculated plants and the EBE predictions, this TALE seems to be able to induce *MeSWEET10a*. However, despite the reduced ability of CFBP1851 to cause symptoms, it still induces water-soaked lesions, indicating that there might be mechanisms that do not rely on *MeSWEET10a* transcriptional activation to promote water soaking, yet less efficiently.

As seen in Southern blot analysis, *Xpm* TALomes are characterized by a limited and bimodal range of variant sizes. This bimodal size distribution could be explained by a recent acquisition of TALEs from two different ancestors or the fixation of two useful alleles that then diverged. Our data favors the second hypothesis since the repeat-based phylogeny (Supplementary Figure S3) shows two clades that group TALEs by size, both emerging from the TALE13 variants. This is probably why there are shared repeat block patterns between the two TALE size groups (Figure 3). Ferreira and collaborators [80] created a TALE-based phylogeny with 122 coding sequences from *Xanthomonas* spp. TALEs. *Xanthomonas phaseoli* pv. *phaseoli* and *Xanthomonas citri* pv. *fuscans*, two pathovars closely related to *Xpm*, show unimodal and continuous TALE size distribution that ranges from 18 to 23 repeats [81]. N-, C-terminal and TALE flanking region analysis suggests that these TALE variants come from a unique common ancestor [81]. *X. campestris* (*Xc*) exhibits a more complex TALE size distribution which however seems to be even from 11 to 22 repeats. Yet, analysis of N-, C-terminal and repeats indicate that *Xc* TALEs emerged from two different ancestors [82], where smaller TALEs (variants with 12, 14 and 15 repeats) have structurally different repeats from larger TALEs with 21 and 22 repeats. Since N- and C-terminal sequences are not available in our study and repeat structure is highly homogeneous, we could not perform such analyses.

TALE sequence diversity was assessed by potential DNA-binding affinity using FuncTAL [61], resulting in five DNA affinity groups and four orphan (non-clustered) effectors. Among them, there are several examples of deletion/duplication, like the case of TALE22D from cluster 1 (discussed earlier), TALE14B in the cluster 3 and TALE13A when compared to TALE14E (see Figure 3). Repeat recombination patterns are also observed; for example, TALE14A variant shows the most common starting RVD block NI-NG-NI-NN, but the remaining block of ten RVDs can be found in TALE22A and B variants. Repeat swaps (non-synonymous point mutations affecting the RVD) seem to be a key variability driver for TALE diversity in *Xpm*, since all the clusters show at least two variants with one to four swaps (see Figure 3). These evolutionary mechanisms for TALEs have been well documented [83,84], and several examples of deletion/duplication, repeat swap and recombination can be found among *Xanthomonas* spp. TALEs [6,81,82].

To elucidate the effects of this functional diversity, we selected two strains whose TALomes covered most of the TALE diversity. Strains UA681 and UA1061 were inoculated in 60,444 varieties to obtain a transcriptomic snapshot of the infection. Briefly, plant response to infection with either of the strains shows a marked alteration of oxidoreductase activity, DNA-transcription factor activity, interaction with metals such as iron and copper, and carbon catabolism. Muñoz-Bodnar and collaborators compared the transcriptomic profiles of cassava plants from the variety MCOL2215 inoculated with two *Xpm* strains with contrasting virulence [41]. In that study, *Xpm* transcriptomic profiles did not significantly differ and showed a transcriptional alteration of genes related to catabolic processes, cellular glucan metabolism, transcription regulation and response to oxidative stress, among other categories. A transcriptomic study in *Xanthomonas oryzae* pv. *oryzicola* (*Xoc*) showed altered expression of genes involved in catalytic and oxidoreductase activities, which suggest an active reactive oxygen species detoxification and cellular redox status control [3]. Taken together, this indicates that alteration of the redox environment and catabolic processes are common to the compatible interactions between *Xanthomonas* spp. and their hosts, highlighting their relevance in pathogenesis.

The analysis of the DUGs coupled to EBE predictions resulted in several candidates that could be important for *Xpm* pathogenesis. Some of these candidate genes were previously validated as actual targets of *Xpm* TALEs (see Table 4), which supports our findings. The sugar transporter encoding *MeSWEET10a* is a DUG highly overexpressed in both treatments (UA681 and UA1061), and it has been shown to play a key role in symptom formation and bacterial growth *in planta* [8]. Our data provide indirect evidence that the new TALE20-derived TALE22D variant is functional and capable of targeting this important *S* gene. In this regard, we explored if the addition of these two repeats at the beginning would negatively affect the predicted DNA-binding affinity on the already known EBE (EBE for TAL20: TATAAACGCTTCTCGCCCATC). Results show that the two nucleotides located right before the EBE for TAL20 are the same first two nucleotides of the EBE (EBE for TAL22D: TATATAAACGCTTCTCGCCCATC), allowing then a perfect match for the new variant. We foresee that these changes might affect TALE-driven induction dynamics, but it is difficult to establish if these will result beneficial for the molecular interaction. Repeats located near to the N-terminal region contribute more to the specific DNA recognition and are more important to the overall affinity of the TALE-DNA interaction [85,86], but, paradoxically, increasing the number of repeats results in lower specificity due to more off-target activation [87]. What implications this change could have in the intensity of binding induction of the gene or in the adaptation of the pathogen to mutations in the promoter needs to be determined. A comparison of *MeSWEET10a* induction in plants treated with strains expressing these two TALE variants would indicate if this mutation has a significant impact on transcriptional activity of the S gene, and the search for this novel variant among populations would shed light on evolutionary dynamics of major TALEs.

The ammonium transporter encoding gene *Manes.08G141800* is one of the most interesting candidates targeted by this new TALE variant. Grewal and coworkers demonstrated that rice response after a few hours of being inoculated with *Xanthomonas oryzae* pv. *oryzae*, a vascular pathogen responsible for the bacterial leaf blight in rice, encompasses the transcriptional alteration of several transporters, including ammonium transporters. Activity of these transporters change ion fluxes and affect the membrane potential, disturbing uptake of other channels and altering activation of defense response. In this regard, they point out that ammonium transporter activity affects K^+^ uptake by cells, which would lead to modified cation balances [88]. It is possible that these ammonium transporters have an analogous effect to that hypothesized for the sulfate transporter coding gene *OsSULTR3;*6 induced by Tal2g in the *Xoc*-rice pathosystem [3]. The expression of these genes might allow the bacteria to alter antioxidant capacity through ion leaking, which would result in interference with redox signaling and defense or in the promotion of water soaking through alteration of osmotic equilibrium.

Functional redundancy by convergence has been demonstrated for TALEs in other pathosystems. In those cases, important susceptibility genes are targeted by two or more TALEs that have potentially evolved from different ancestors and converge on EBEs present in the same promoter region [1,7,53,89,90,91]. Our data suggested that candidate genes *Manes.04G033900* and *Manes.13G045100* are targeted by TALE22 variants (which was already validated by Cohn and coworkers [8]) and also by the TALE21A variant found in CFBP1851, through overlapped EBEs. Our RT-PCR results indicate that *Manes.13G045100* is actually upregulated *in planta* after inoculation with CFBP1851 strain (Figure 7B), showing that this is a potential case of functional convergence and a possible relevant target for the pathogen. However, even if at a first glance TALE21A variant has important structural differences when compared to TALE22 variants, DisTAL analysis indicates that variant TALE21A is very close to TALE22 variants, which would reflect the history of close evolving effectors that maintain affinities. *Manes.13G045100* encodes for a CLAVATA3/ESR (CLE) peptide, a protein family of ligands involved in definition of cell fate with a key role in cellular division control in the shoot apical meristem [92,93]. This ligand family has been shown to be important in symbiotic processes through the regulation of nodule formation, and in pathogenic interactions where phytopathogens hijack the CLE-mediated developmental signaling pathway to improve fitness and colonization rate (reviewed by [94]). Nematodes *Globodera rostochiensis* [95] and *Heterodera glycines* [96] secrete effectors that mimic CLE functions which allow them to reprogram cell growth at the point of infection. To our knowledge, there are no reports of phytopathogenic bacteria using this pathway as a virulence factor, but there are other examples of cell fate-related transcriptional factors, *UPA20* and *UPA7* [12,97], which are also transcriptionally activated by TALEs (AvrBs3). Taking all this into consideration, validation and further studies on this TALE target could shed light on new host susceptibility pathways.

## 5. Conclusions

This work describes for the first time the TALE variability on *Xpm* populations. TALome sequences allowed us to make some evolutionary and functional inferences on TALE biology for the *Xpm*-cassava pathosystem. Functional clustering of TALEs unveiled traces of recombination, repeat swaps and repeat duplication/deletion. Transcriptomics indicate that new TALE20-derived TALE22D variant preserves the ability to activate the *S* gene *MeSWEET10a*. RNAseq with two *Xpm* strains showed that common up- and down-regulated genes have virtually the same behavior in terms of expression magnitude and regulation, and differentially expressed genes fall consistently in oxidoreductase and catabolic activity categories. Our results pinpoint new potential TALE targets that could have a role in pathogenesis. Further studies are needed to validate the TALE-dependent activation of these targets through the use of mutant strains inactivated in these TALEs and characterize their role during infection using artificial TALEs.

## Figures and Tables

**Table 1:** Primers used for RT-qPCR assays.

Target Gene ^a^	Type	Sequence (5′→3′)	Reference
Candidate gene: Manes.06G123400 (*MeSWEET10a*)	Fw	TCCTCACCTTGACTGCGGTG	[8]
	Rv	AGCACCATCTGGACAATCCCA	
Candidate gene: Manes.04G033900 (Dof domain, zinc finger)	Fw	AAAGTGCCCAAGAGGTGGTG	This study
	Rv	GCCTTTCACTTGAAGCTGGG	
Candidate gene: Manes.13G045100 (Clavata3/ESR CLE-related protein)	Fw	CCACGACGAACTTTCACCCA	This study
	Rv	CGCTGGGAACTTCATGAGCT	
Candidate gene: Manes.11G151300 (Serine carboxypeptidase)	Fw	GCCCCAACTGTTAGATTTGTGG	This study
	Rv	GGTGACCAGCTTCATACACCTT	
Candidate gene: Manes.15G052000 (Beta-glucosidase)	Fw	TTGAAGATATGCTCAACGACACG	This study
	Rv	CGTCTGCTCCGTTCCTGATA	
Reference gene: Manes.08G061700.1 (Tubulin beta-6 chain)	Fw	GGAAAGATGAGCACCAAGGA	[56]
	Rv	ACCAGTATACCAGTGCAAGAAG	

^a^ Gene ID for Phytozome’s Cassava genome annotation version 6.1, main functional annotation is indicated in parentheses.

**Table 2:** General characteristics of the *Xpm* strains selected in this study.

Strain	Region of Origin (ECZ ^a^)	Location of Origin	Year of Isolation	AFLP-Based Haplotype ^b^	Estimated Number of TALEs ^c^
CFBP1851	Caribbean coast (ECZ 1)	nd	1974	3 *	2
CIO151	Eastern plains (ECZ 2)	Meta	1995	3 *	5
UA226	Caribbean coast (ECZ1)	Chinú	2008	5 *	3
UA306	Caribbean coast (ECZ 1)	Palmitos	2008	7 *	4
UA318	Caribbean coast (ECZ 1)	Ciénaga de oro	2008	3 *	4
UA522	Caribbean coast (ECZ 1)	Chinú	2009	4 *	5
UA531	Caribbean coast (ECZ 1)	Chinú	2009	5 *	3
UA681	Caribbean coast (ECZ 1)	Chinú	2009	8 *	5
UA1061	Caribbean coast (ECZ 1)	Chinú	2009	5 *	2
UA1069	Caribbean coast (ECZ 1)	Chinú	2009	5 *	2
UA1183	Eastern plains (ECZ 2)	Villavicencio	2011	1 **	5
UA1211	Eastern plains (ECZ 2)	Granada	2011	2 **	5
UA1235	Eastern plains (ECZ 2)	Villavicencio	2011	3 **	5
UA1245	Eastern plains (ECZ 2)	Villavicencio	2011	3 **	5
UA1357	Eastern plains (ECZ 2)	Orocué	2012	8 **	4
UA1381	Eastern plains (ECZ 2)	Orocué	2012	6 **	4
UA1396	Eastern plains (ECZ 2)	Orocué	2012	7 **	5
UA1399	Eastern plains (ECZ 2)	Orocué	2012	7 **	5

^a^ Previously described in [35]. ^b^ Numbers were arbitrarily assigned to clades of distance trees from data reported by [49] (*) or [50] (**). Since analyses were performed separately, clade numbers from [49,50] are not shared. ^c^ Number of TALE bands determined by RFLP. nd, no data.

**Table 3:** Translated RVD strings of the TALE gene variants sequenced in this study.

Variant	RVD Sequence	Occurrences	Strains
TALE13A	NI-NS-NN-HD-NG-HD-NI-NG-HD-NN-NI-NI-NG	9	UA306, UA318, UA522, UA681, UA1211, UA1235, UA1245, UA1357, UA1399
TALE14A	NI-NG-NI-NN-HD-HD-NG-NG-HD-NG-NG-HD-NG-NG	1	CIO151
TALE14B	NI-NG-NI-NN-HD-HD-NS-NS-NS-HD-HD-NS-HD-NG	1	UA318
TALE14C	NI-NG-NI-NN-NG-HD-NS-NS-NN-NG-HD-NN-NI-NG	9	UA226, UA306, UA318, UA522, UA531, UA681, UA1061, UA1183, UA1381
TALE14D	NI-NG-NI-NN-NI-HD-NS-NS-NN-NG-HD-NN-NI-NG	1	CFBP1851
TALE14E	NI-NS-NN-HD-NG-HD-NI-NS-NN-HD-NN-NI-NI-NG	4	UA226, UA531, UA1061, UA1069
TALE15A	NI-NG-NI-NN-HD-HD-NS-NS-NS-HD-HD-NN-HD-NG-NG	1	UA1211
TALE15B	NI-NG-NI-NN-HD-HD-NS-NS-NS-HD-HD-NS-HD-NG-NG	3	UA522, UA681, UA1235
TALE20A	NI-NG-NI-NN-HD-HD-NN-HD-NN-NG-HD-NS-HD-NN-HD-NG-HD-NI-NG-NG	1	UA318
TALE20B	NI-NG-NI-NN-NG-HD-NN-HD-NN-NG-HD-NG-HD-NN-HD-NG-HD-NN-NG-NG	2	UA226, UA531
TALE20C	NI-NG-NI-NN-NG-HD-NN-HD-NN-NG-HD-NS-HD-NN-HD-NG-HD-NI-NG-NG	3	UA306, UA522, UA681
TALE20D	NI-NG-NI-NN-NG-HD-NS-NS-NN-NG-HD-NG-HD-NN-HD-HD-NS-NI-NG-NG	1	UA1381
TALE20E	NI-NG-NI-NN-NG-HD-NS-NS-NN-NG-HD-NS-HD-NN-HD-NN-HD-NI-NS-NG	1	UA1235
TALE21A	NI-NG-HD-NG-HD-N*-NG-NG-HD-HD-NG-NG-N*-NG-HD-NG-NG-NG-HD-NG-NG	1	CFBP1851
TALE22A	NI-NG-HD-NG-NG-NG-HD-HD-NG-NG-HD-NG-HD-HD-NG-NG-HD-NG-NG-HD-NG-NG	1	UA1235
TALE22B	NI-NG-HD-NG-NG-NG-HD-HD-NG-NG-NG-NG-HD-HD-NG-NG-HD-NG-NG-HD-NG-NG	4	UA306, UA318, UA522, UA681
TALE22C	NI-NG-HD-NG-NG-NG-HD-HD-NG-NG-NG-NN-HD-NN-NG-NG-HD-NG-NG-HD-NG-NG	2	UA531, UA1061
TALE22D	NI-NG-NI-NG-NI-NN-NG-HD-NN-HD-NN-NG-HD-NG-HD-NN-HD-NG-HD-NN-NG-NG	1	UA1061

**Table 4:** Candidate gene targets for TALEs present in strains UA681 and UA1061 with good to excellent EBE predictions.

	Target Gene ID ^a^	Annotations ^a^	EBE Prediction Quality ^c^								Retained After Second Filter? ^e^	Responsible for Activation (Ref)
			T13A (NC ^b^)	T14E (NC ^b^)	T14C (C4 ^b^)	T15B (C3 ^b^)	T20C (C1 ^b^)	T22D (C1 ^b^)	T22B (C2 ^b^)	T22C (C2 ^b^)		
Common DUGs	Manes.04G033900	Dof Domain, Zinc Finger Protein							E	E	Yes	TAL22 [8]
	Manes.06G123400	Bidirectional Sugar Transporter Sweet10					VG	E			Yes	TAL20 [8]
	Manes.17G096100	No Data					VG	VG			No	
	Manes.15G026800	Membrane-Associated Kinase Regulator			E						Yes	TAL14 [8]
	Manes.10G007900	Abscisic Acid Receptor PYL4							G	F	Yes	
	Manes.11G151300	Serine Carboxypeptidase S10					F	G			Yes	TAL14 [8]
DUGs for UA681	Manes.15G041200	12s Seed Storage Protein							VG		No	
	Manes.18G060400	Rho GTPase-Activating Protein Ren1							E		Yes	
	Manes.13G045100	Clavata3/ESR (CLE)-Related Protein							E		Yes	TAL22 [8]
	Manes.S045100	Class IV Chitinase					E				Yes	
	Manes.04G109300	Oxidoreductase, 2OG-Fe (II) Oxygenase Family Protein				F	VG				No	
	Manes.06G159100	Ring Finger Domain (ZF-Ring_2)/Wall-Associated Receptor Kinase C-Terminal	F			G					No	
	Manes.09G134100	mlo Protein				E					No	
	Manes.14G044700	Protein Containing an AP2 Domain			F	E					Yes	TAL15 [8]
	Manes.04G096600	Xyloglucan:Xyloglucosyl Transferase			VG ^d^						No	
	Manes.17G038600	No Data			E						No	
	Manes.05G118500	No Data	G			F	F				No	
	Manes.02G120700	Protein Containing a Tetratricopeptide Repeat (TPR_16)	G								No	
	Manes.12G151000	No Data	VG								Yes	
	Manes.09G105800	Protein Containing a Myb-Like DNA-Binding Domain and a Myb-CC Type Transfactor, LHEQLE Motif					G				Yes	
	Manes.18G074600	No Data					G				No	
	Manes.S013800	Basic Secretory Protein Family/Peptidase of Plants and Bacteria					G ^d^				No	
	Manes.06G057400	Equilibrative Nucleoside Transporter							G		No	
	Manes.07G135200	Abscisic Acid Receptor PYL4							G		Yes	
	Manes.04G053400	Galactolipase/Phospholipase A (1)				F			G		No	
	Manes.08G036300	Calcium-Dependent Protein Kinase				G					Yes	
DUGs for UA1061	Manes.07G122400	No Data								VG	Yes	
	Manes.08G141800	Ammonium Transporter 1						E			Yes	
	Manes.11G008300	Member Of ‘GDXG’ Family of Lipolytic Enzymes						VG			Yes	
	Manes.10G066600	2-Hydroxyisoflavanone Dehydratase						E			Yes	
	Manes.16G117500	No Data			VG						Yes	
	Manes.13G136600	Coniferyl-Alcohol Glucosyltransferase			E						No	
	Manes.04G118200	Protein of Unknown Function (Duf642)		G ^d^							Yes	
	Manes.15G090000	Homeobox-Leucine Zipper Protein ATHB-52						G			Yes	TAL14 [8]

^a^ Gene identifiers and annotations were extracted from Phytozome’s cassava genome version 6.1. ^b^ T stands for TALE. The bracketed alphanumeric code corresponds to the cluster number derived from FuncTAL analysis (Section 3.3 and Figure 3). NC, non-clustered; C1 to C4, cluster 1 through 4. ^c^ The quality of EBE prediction for a given candidate was calculated as the sum of the four determined ranks (one per software). Totals were then categorized as E (Excellent, combined rank = 1 to 400), VG (Very Good, combined rank = 401 to 800), G (Good, combined rank = 801 to 1200), and F (Fair, combined rank = 1201 to 1600). ^d^ These EBE predictions were not found in the corresponding 60444 promoter sequence. ^e^ EBE distance to TSS/TLS between 152 bp and 63 bp.

## Data Availability

Publicly available datasets were analyzed in this study. TALE nucleotide sequences can be found at GenBank under accession numbers MW413757-MW413802. RNA-Seq data can be found in the SRA database under the BioProject No. PRJNA688032, BioSample No. SAMN17167922 (https://www.ncbi.nlm.nih.gov/sra/PRJNA688032).

## Supplementary Materials

The following are available online at https://www.mdpi.com/2076-2607/9/2/315/s1, Figure S1: Two edaphoclimatic zones (ECZs) where cassava is grown in Colombia and strain origins. The blue region corresponds to the Caribbean coast ECZ delimited as two departments: Córdoba and Sucre. The yellow region corresponds to the Eastern plains ECZ delimited as two departments: Casanare and Meta. Black dots show municipalities where strains were collected. The list of strains from each ECZ includes the collection date in parentheses. Figure S2: Southern blots for the characterized strains. The upper panel shows lanes for strains from the Caribbean coast and strains isolated from the department Meta (Eastern plains). The lower panel shows lanes for the strains from the department Casanare (Eastern plains). The ID of each strain is marked above each lane. MWM = molecular weight marker; TALE1_Xam_ = pF3 vector containing TALE1_Xam_. This vector was also digested with *BamHI* and loaded into each gel as a positive control and size indicator. The molecular weight marker is presented as seen in the stained gel to estimate sizes. Figure S3: Repeat-based phylogeny created by DisTAL. Unrooted cladogram showing the phylogenetic relationship among nucleotide variants according to nucleotide composition of repeats after masking positions coding for RVDs. Labels indicate the variant classification according to the RVD string (T stands for TALE) and the strain where the gene was isolated. Colors are related to the clusters defined in Figure 3: blue = Cluster 1, magenta = Cluster 2, red = Cluster 3, cyan = Cluster 4, yellow = Cluster 5; orphans are all in black. Values on branches correspond to distances obtained from pair-wise alignments for repeat-based coded TALEs calculated by DisTAL. Figure S4: TALE-targeted candidate prediction for A) UA681 and B) UA1061 DUGs. These heatmaps show the best optioned candidates and their EBE-prediction quality from each software used. Top labels indicate the TALE variant that is predicted to bind each gene and the DNA-binding affinity cluster where it belongs. Rank indicates the position of the prediction (which is a reflect of the score) among the set of the top 400 predictions. Genes over the dashed horizontal line are considered as the best optioned candidates (see text). Figure S5: Selective GO-term enrichment analysis showing the cellular component (CC), molecular function (MF) and biological process (BP) categories that are significantly enriched with DEGs carrying EBEs on their promoters in UA681- or UA1061-treated plants. Discontinuous lines show the *p*-value thresholds corresponding to 0.05 (blue) and 0.01 (dark gray). Table S1. EBE position comparison between the promoter context of genes reported for varieties AM560-2 and 60444 genomes. Table S2. Potential cases of functional convergence among DUGs with predicted EBEs. Candidates are sorted from the top to the bottom according to the EBE prediction quality. Table S3. ID and short description of GO Terms displayed on Figure 4 and Figure S5. The order of GO Terms matches the order displayed on the *y*-axis of both figures.
