# Supplementary material for: TAL Effector Repertoires of Strains of Xanthomonas phaseoli pv. manihotis in Commercial Cassava Crops Reveal High Diversity at the Country Scale
Source: Microorganisms. 2021 Feb 4;9(2):315. doi: 10.3390/microorganisms9020315 (PMC7913752; doi:10.3390/microorganisms9020315)
Supplement: Supplementary file 1 [file microorganisms-09-00315-s001.pdf]

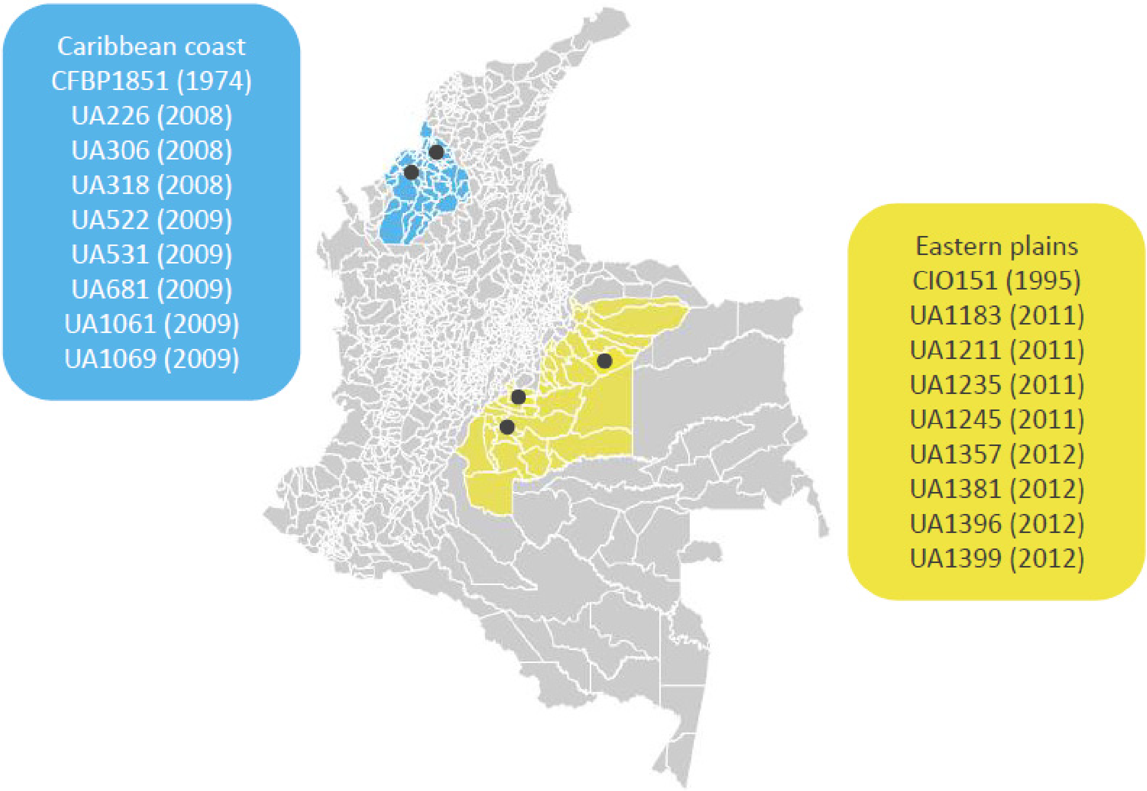

Figure S1: Two edaphoclimatic zones (ECZs) where cassava is grown in Colombia and strain origins. The blue region corresponds to the Caribbean coast ECZ delimited as two departments: Córdoba and Sucre. The yellow region corresponds to the Eastern plains ECZ delimited as two departments: Casanare and Meta. Black dots show municipalities where strains were collected. The list of strains from each ECZ includes the collection date in parentheses.

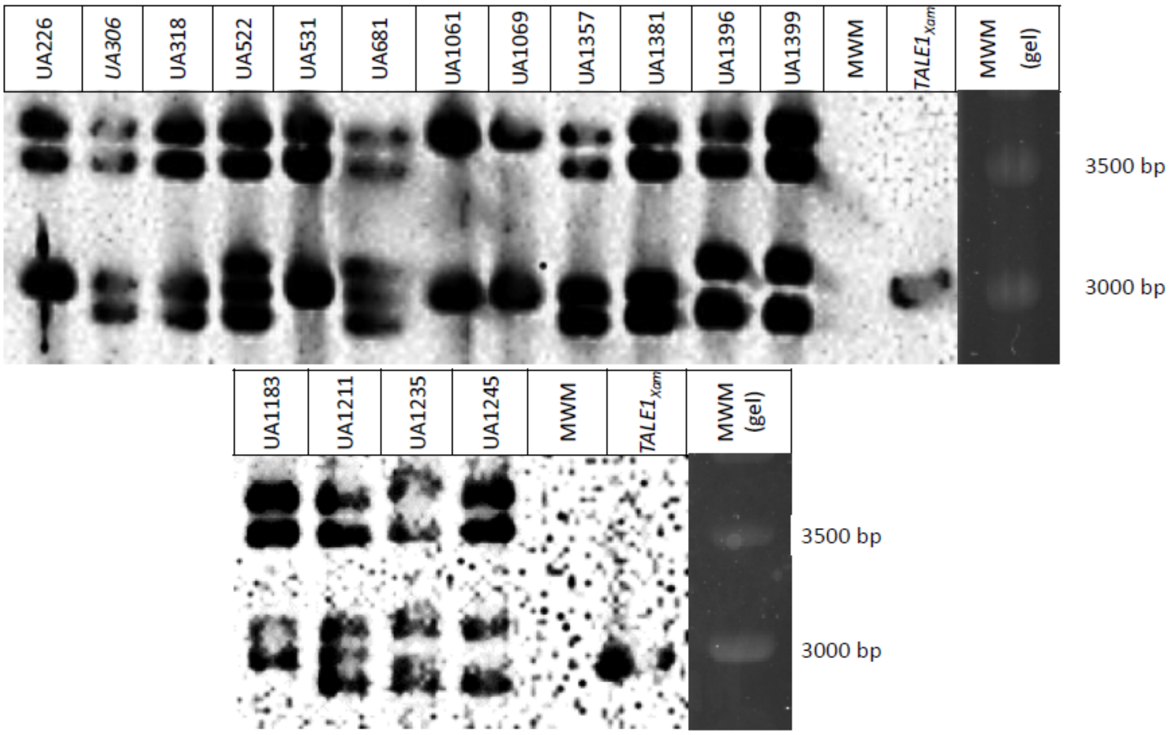

Figure S2: Southern blots for the characterized strains. The upper panel shows lanes for strains from the Caribbean coast and strains isolated from the department Meta (Eastern plains). The lower panel shows lanes for the strains from the department Casanare (Eastern plains). The ID of each strain is marked above each lane. MWM = molecular weight marker; *TALE1Xam* = pF3 vector containing *TALE1Xam*. This vector was also digested with *BamHI* and loaded into each gel as a positive control and size indicator. The molecular weight marker is presented as seen in the stained gel to estimate sizes.

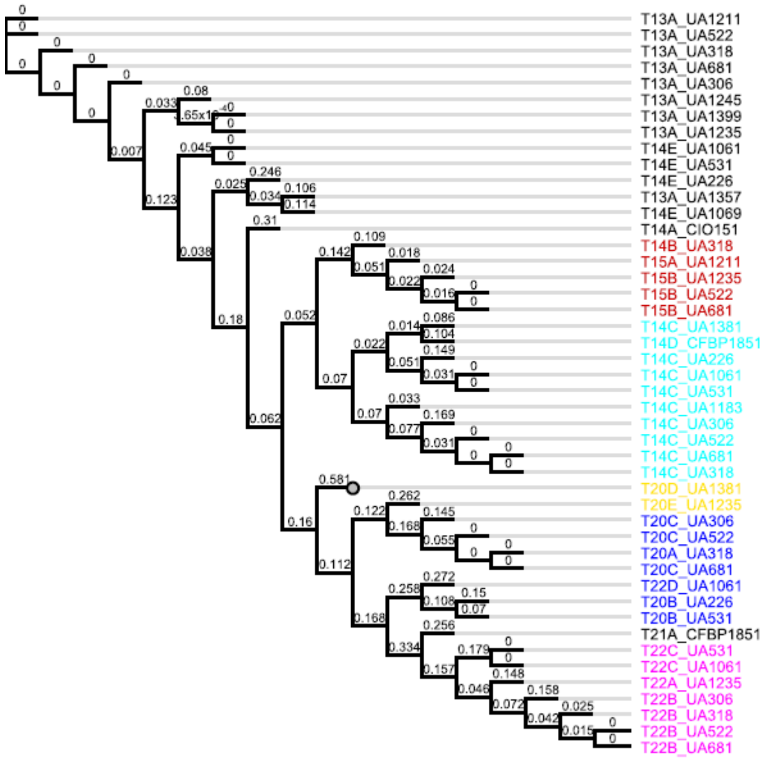

Figure S3: Repeat-based phylogeny created by DisTAL. Unrooted cladogram showing the phylogenetic relationship among nucleotide variants according to nucleotide composition of repeats after masking positions coding for RVDs. Labels indicate the variant classification according to the RVD string (T stands for TALE) and the strain where the gene was isolated. Colors are related to the clusters defined in Figure 3: blue = Cluster 1, magenta = Cluster 2, red = Cluster 3, cyan = Cluster 4, yellow = Cluster 5; orphans are all in black. Values on branches correspond to distances obtained from pair-wise alignments for repeat-based coded TALEs calculated by DisTAL.

A)

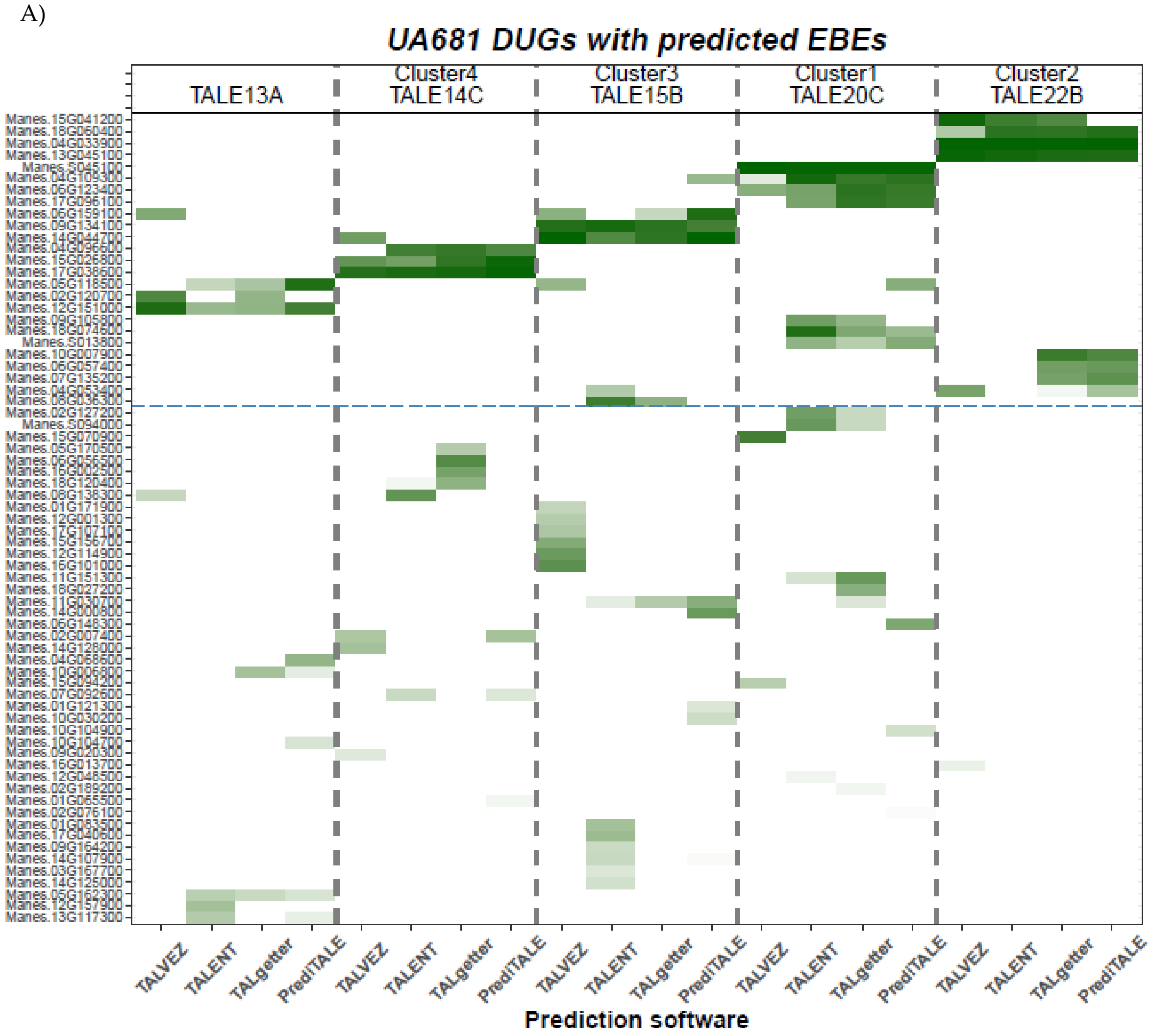
UA681 DUGs with predicted EBEs
TALE13A
Cluster4
TALE14C
Cluster3
TALE15B
Cluster1
TALE20C
Cluster2
TALE22B
TALVEZ
TALENT
TALgetter
PrediTALE
Prediction software

B)

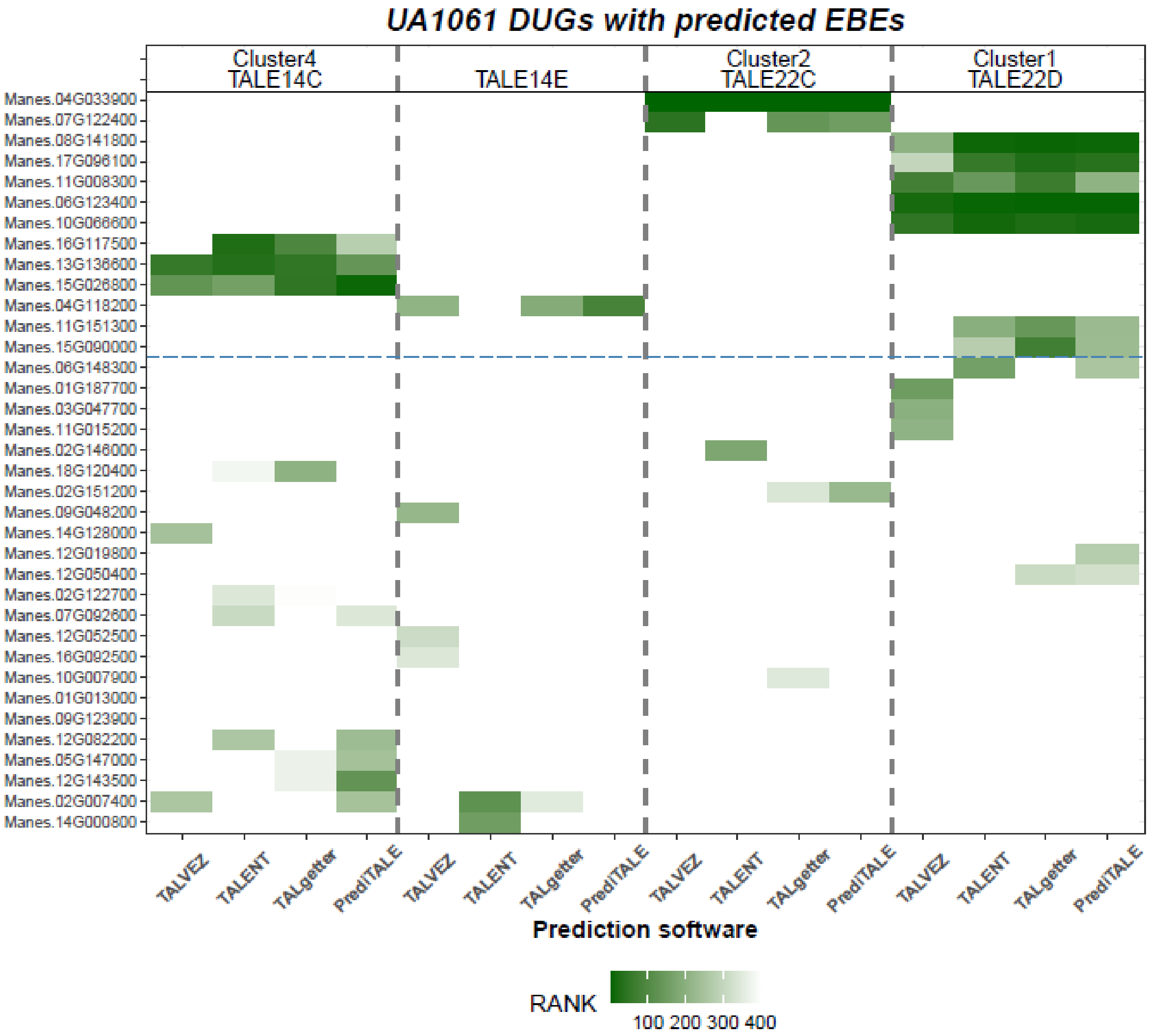

Figure S4: TALE-targeted candidate prediction for A) UA681 and B) UA1061 DUGs. These heatmaps show the best optioned candidates and their EBE-prediction quality from each software used. Top labels indicate the TALE variant that is predicted to bind each gene and the DNA-binding affinity cluster where it belongs. Rank indicates the position of the prediction (which is a reflect of the score) among the set of the top 400 predictions. Genes over the dashed horizontal line are considered as the best optioned candidates (see text)

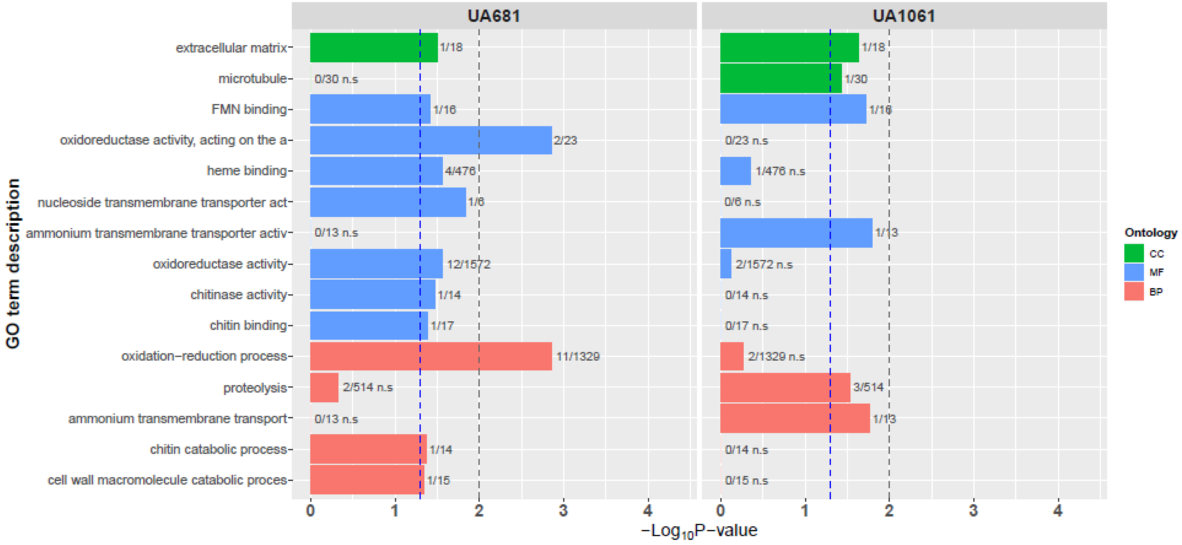

Figure S5: Selective GO-term enrichment analysis showing the cellular component (CC), molecular function (MF) and biological process (BP) categories that are significantly enriched with DEGs carrying EBEs on their promoters in UA681- or UA1061-treated plants. Discontinuous lines show the p-value thresholds corresponding to 0.05 (blue) and 0.01 (dark gray).

Table S1. EBE position comparison between the promoter context of genes reported for varieties AM560-2 and 60444 genomes.

Target Gene ID[a]	TALE ID[b]	EBE Sequence	60444 EBE search	60444 EBE position[c]	AM560-2 EBE position[b]
Manes.02G120700	T13A	ATCCGAGGAGCTTA	Exact match	-463	-463
Manes.04G033900	T22B	TATCTTTCCTTCACCTTCTTCTT	Exact match	-169	-168
Manes.04G053400	T15B	TATAAACCAATCACTT	Exact match	-91	-91
Manes.04G053400	T22B	CATCTTTTCTTTCCCTTCTACTC	Exact match	-162	-162
Manes.04G096600	T14C	ATTTACTTGATTATA	Not found	NA	-823
Manes.04G109300	T20C	AATGATTTGGATGTGTTTATA	Exact match	-355	-855
Manes.05G118500	T13A	TAGGATCATCAAAT	Exact match	-538	-537
Manes.05G118500	T20C	AATTAATGCAATGTTACTATA	Exact match	-514	-513
Manes.05G118500	T15B	TCTCCCCCAACCGCCT	Exact match	-94	-94
Manes.06G057400	T22B	AATCTTTTCTTTTTCTTCTCCTT	Exact match	-716	-731
Manes.06G123400	T20C	TATAAACGCTTCTCGCCCATC	Exact match	-109	-109
Manes.06G123400	T22D	TATATAAACGCTTCTCGCCCATC	Exact match	-111	-111
Manes.06G159100	T15B	TATAGCCTCACCGCAT	Exact match	-887	-887
Manes.06G159100	T13A	TAGCCTCACCGCAT	Exact match	-885	-885
Manes.07G135200	T22B	TTTCTTTTCTTTTCCTTCTCTTT	Imperfect match	-135	-126
Manes.08G036300	T15B	ATGGGGTTTTGTTATA	Exact match	-47	-47
Manes.08G141800	T22D	TAAATACTCACCTCTTGCTCTTT	Exact match	-37	-37
Manes.09G105800	T20C	TATAATCCTATAATCCTCATT	Imperfect match	-76	-76
Manes.09G134100	T15B	TCTAACCAACCCCCTT	Exact match	-976	-313
Manes.10G007900	T22B	TTTCCTTCCTTTTCCTTCTCTTT	Exact match	-56	-56
Manes.10G066600	T22D	TATAAACTCACGTCTCAATCGCA	Exact match	-94	-94
Manes.11G008300	T22D	TATAAAGTCATGCCTAGCCCCTT	Exact match	-78	-78
Manes.11G151300	T22D	TATATAAACCAGTCAAGATCCTT	Exact match	-72	-72
Manes.11G151300	T20C	TATAAACCAGTCAAGATCCTT	Exact match	-70	-70
Manes.12G151000	T13A	ATGTGATGAGCCTA	Exact match	-80	-80

Manes.13G045100	T22B	TCTATTTCCCTTTCCTTCTCCTT	Exact match	-48	-48
Manes.13G136600	T14C	TACAGTCAAATCAAT	Exact match	-253	-253
Manes.14G044700	T14C	TCTACTCAGCTCGAT	Exact match	-25	-25
Manes.15G026800	T14C	TATAATCATATCGAT	Exact match	-95	-93
Manes.15G041200	T22B	AAGGGGAAGGAGAAGGAGAGAGA	Exact match	-629	-629
Manes.15G041200	T22B	AAGAGGAAGGGGAAGGAGAAGGA	Exact match	-635	-635
Manes.15G090000	T22D	TAATTAGCCGCCTCTCCCTCTCT	Exact match	-150	-150
Manes.16G117500	T14C	ATTGTCTTGATTATA	Exact match	-464	-464
Manes.17G038600	T14C	TATAGTCCACTCAAT	Imperfect match	-751	-762
Manes.17G096100	T22D	TATATAACCGTCTCGCGCTCTTT	Exact match	-352	-352
Manes.17G096100	T20C	TATAACCGTCTCGCGCTCTTT	Exact match	-350	-350
Manes.18G060400	T22B	TATCTTTACGCTTCCTTCTCCTT	Exact match	-232	-232
Manes.18G074600	T20C	AATCAGTGTGATTTTAGTATA	Exact match	-244	-234
Manes.S013800	T20C	TATAATTAAATCATGATCATT	Not found	NA	-860
Manes.S045100	T20C	TCCAGTCACATCCCACTCATT	Exact match	-42	-42

[a] Gene identifiers and annotations were extracted from Phytozome's cassava genome version 6.1. [b]T stands for TALE. [c]Position calculated from the first nucleotide of the EBE to the start codon of each gene.

Table S2. Potential cases of functional convergence among DUGs with predicted EBEs. Candidates are sorted from the top to the bottom according to the EBE prediction quality.

Target gene ID[a]	Annotations[a]	Potentially convergent clusters or TALEs
Manes.04G033900	Dof Domain, Zinc Finger Protein	Cluster 2 (TALE22 variants) and TALE21A
Manes.13G045100	Clavata3/ESR (CLE)-Related Protein	Cluster 2 (TALE22 variants) and TALE21A
Manes.14G044700	Protein Containing an AP2 Domain	Cluster 3 (TALE15 variants) and Cluster 4 (TALE14 variants)
Manes.05G118500	No Data	Cluster 3 (TALE15 variants) and TALE13A
Manes.06G159100	Ring Finger Domain (ZF-Ring_2)/Wall-Associated Receptor Kinase C-Terminal	Cluster 3 (TALE15 variants) and TALE13A
Manes.04G109300	Oxidoreductase, 2OG-Fe (II) Oxygenase Family Protein	Cluster 1 (TALE20 variants) and Cluster 3 (TALE15 variants)
Manes.04G053400	Galactolipase/Phospholipase A(1)	Cluster 2 (TALE22 variants) and Cluster 3 (TALE15 variants)
Manes.16G117500	No Data	Cluster 4 (TALE14 variants) and TALE14E

[a] Gene identifiers and annotations were extracted from Phytozome's cassava genome version 6.1.

Table S3. ID and short description of GO Terms displayed on Figures 4 and S5. The order of GO Terms matches the order displayed on the y-axis of both figures.

Go Term ID	GO Term	Source
GO:0031012	extracellular matrix	Figure 4
GO:0048046	apoplast	Figure 4
GO:0005618	cell wall	Figure 4
GO:0016020	membrane	Figure 4
GO:0020037	heme binding	Figure 4
GO:0005506	iron ion binding	Figure 4
GO:0016491	oxidoreductase activity	Figure 4
GO:0016705	oxidoreductase activity, acting on paired donors, with incorporation or reduction of molecular oxygen	Figure 4
GO:0004553	hydrolase activity, hydrolyzing O-glycosyl compounds	Figure 4
GO:0003700	DNA-binding transcription factor activity	Figure 4
GO:0016758	transferase activity, transferring hexosyl groups	Figure 4
GO:0008519	ammonium transmembrane transporter activity	Figure 4
GO:0016762	xyloglucan:xyloglucosyl transferase activity	Figure 4
GO:0004222	metalloendopeptidase activity	Figure 4
GO:0005507	copper ion binding	Figure 4
GO:0004601	peroxidase activity	Figure 4

GO:0016887	ATPase activity	Figure 4
GO:0043565	sequence-specific DNA binding	Figure 4
GO:0016757	transferase activity, transferring glycosyl groups	Figure 4
GO:0055114	oxidation-reduction process	Figure 4
GO:0008152	metabolic process	Figure 4
GO:0048544	recognition of pollen	Figure 4
GO:0072488	ammonium transmembrane transport	Figure 4
GO:0006073	cellular glucan metabolic process	Figure 4
GO:0031012	extracellular matrix	Figure S5
GO:0005874	microtubule	Figure S5
GO:0016620	oxidoreductase activity, acting on the aldehyde or oxo group of donors, NAD or NADP as acceptor	Figure S5
GO:0005337	nucleoside transmembrane transporter activity	Figure S5
GO:0008519	ammonium transmembrane transporter activity	Figure S5
GO:0010181	FMN binding	Figure S5
GO:0020037	heme binding	Figure S5
GO:0016491	oxidoreductase activity	Figure S5
GO:0004568	chitinase activity	Figure S5
GO:0008061	chitin binding	Figure S5
GO:0055114	oxidation-reduction process	Figure S5
GO:0072488	ammonium transmembrane transport	Figure S5
GO:0006508	proteolysis	Figure S5
GO:0006032	chitin catabolic process	Figure S5
GO:0016998	cell wall macromolecule catabolic process	Figure S5
